# Visualization and Analysis of the Dynamic Assembly of a Heterologous Lantibiotic Biosynthesis Complex in Bacillus subtilis

**DOI:** 10.1128/mBio.01219-21

**Published:** 2021-07-20

**Authors:** Jingqi Chen, Auke J. van Heel, Oscar P. Kuipers

**Affiliations:** a Department of Molecular Genetics, Groningen Biomolecular Sciences and Biotechnology Institute, University of Groningen, Groningen, the Netherlands; Heinrich Heine University; Nanyang Technological University

**Keywords:** *Bacillus subtilis*, nisin, lantibiotic biosynthesis machinery, subcellular localization, assembly dynamics, fluorescence microscopy

## Abstract

A membrane-associated lanthipeptide synthetase complex, consisting of the dehydratase NisB, the cyclase NisC, and the ABC transporter NisT, has been described for nisin biosynthesis in the coccoid bacterium Lactococcus lactis. Here, we used advanced fluorescence microscopy to visualize the functional nisin biosynthesis machinery in rod-shaped cells and analyzed its spatial distribution and dynamics employing a platform we developed for heterologous production of nisin in Bacillus subtilis. We observed that NisT, as well as NisB and NisC, were all distributed in a punctate pattern along the cell periphery, opposed to the situation in coccoid cells. NisBTC proteins were found to be highly colocalized, being visualized at the same spots by dual fluorescence microscopy. In conjunction with the successful isolation of the biosynthetic complex NisBTC from the cell membrane, this corroborated that the visual bright foci were the sites for nisin maturation and transportation. A strategy of differential timing of expression was employed to demonstrate the *in vivo* dynamic assembly of NisBTC, revealing the recruitment by NisT of NisBC to the membrane. Additionally, by use of mutated proteins, the nucleotide binding domain (NBD) of NisT was found to function as a membrane anchor for NisB and/or NisC. We also show that the nisin biosynthesis sites are static and likely associated with proteins residing in lipid rafts. Based on these data, we propose a model for a three-phase production of modified precursor nisin in rod-shaped bacteria, presenting the assembly dynamics of NisBTC and emphasizing the crucial role of NisBC, next to NisT, in the process of precursor nisin translocation.

## INTRODUCTION

Lanthipeptides belong to a family of ribosomally synthesized and posttranslationally modified peptides (RiPPs) containing (methyl-)lanthionine residues. Lantibiotics are lanthipeptides with antimicrobial activity. Lanthipeptides are biosynthesized from a genetically encoded precursor peptide that is composed of a C-terminal core peptide (CP), where the posttranslational modifications occur, and an N-terminal leader peptide (LP), which is often essential for recognition by the modification and transportation machinery and keeping the lantibiotic peptide initially antimicrobially inactive (1). Commonly, lanthipeptides are organized in a biosynthetic gene cluster encoding the precursor peptide (LanA), modification enzymes (either LanB and LanC or LanM/LanKC/LanL), ABC transporter (LanT), protease (LanP), two-component regulation system (LanR and LanK), immunity system (LanI and LanFEG), and accessory proteins (e.g., LanH) (2). According to the biosynthetic machinery responsible for installing the thioether rings, lanthipeptides are subdivided into four different classes (I to IV) (3–6). Recently, lanthidins were proposed to be class V lanthipeptides that are made via a biosynthetically distinct pathway (7, 8).

The mechanism of maturation, transport, immunity, and regulation for lantibiotics has been relatively well understood (Fig. S1A in the supplemental material), and structural data are already available for some of the proteins involved (2, 9–13). Based on previous studies, cytoplasmic membrane-associated multicomponent enzymatic complexes have been proposed for the maturation and transportation of class I and II lanthipeptides (14–18). In class I lanthipeptides, nisin modification enzymes, the dehydratase NisB and the cyclase NisC, were demonstrated to be present in the cytoplasmic membrane (19). Using coimmunoprecipitation and a yeast two-hybrid screen, a molecular interaction between NisB and NisC, as well as NisC and NisT, which exports nisin from the cell, was detected, suggesting the existence of a nisin biosynthesis-associated complex NisBTC in the cell membrane of L. lactis (14). A recent *in vitro* study detected an interaction of NisT with NisB besides the interaction between NisT and NisC (20). For subtilin biosynthesis, SpaB was shown to localize to the cytoplasmic membrane in Bacillus subtilis (21) and interact with SpaC when both proteins were overexpressed in Escherichia coli (22). Moreover, SpaB, SpaC, and SpaT were reported to form a membrane-associated complex SpaBTC in B. subtilis (15). For class II lanthipeptides, the enzyme NukM and the ABC transporter NukT were proven to assemble a membrane-located multimeric protein complex NukMT for the production of Nukacin ISK-1 in Staphylococcus warneri ISK-1 by yeast two-hybrid assays and surface plasmon resonance (SPR). NukM expressed heterologously in Staphylococcus carnosus TM300 was located at the cytoplasmic membrane even in the absence of NukT (18). In spite of these data, direct evidence supporting the presence of a lantibiotic biosynthesis machinery LanBTC associated with the cell membrane remains to be provided. Until now, only few subcomplexes involved in the modification of precursor nisin (NisA) have been characterized. Extensive studies on the complex NisAB yielding structural insight have been reported (9, 23–25). A pulldown assay demonstrated that NisB and NisC could be copurified with an engineered His-tagged NisA (16). The assembly of the complex NisABC was conducted *in vivo*, and the complex was suggested to comprise a NisB dimer, a monomer of NisC, and one precursor nisin (17).

10.1128/mBio.01219-21.1 FIG S1 LanABTC system in bacteria. (A) Biosynthesis, regulation, and immunity of class I lanthipeptides. LanA is a ribosomally synthesized peptide with a leader peptide and a core peptide and is targeted to a putative lanthipeptide biosynthesis machinery consisting of the dehydratase LanB, the cyclase LanC, and the ABC transporter LanT. LanB converts serine and threonine residues into dehydroalanine and dehydrobutyrine, respectively. LanC catalyzes the addition of a thiol group in cysteine to an N-terminally located dehydroamino acid resulting lanthionine rings. LanT exports the fully modified lanthipeptide outside the cells, where the serine protease LanP extracellularly removes the leader peptide releasing active lanthipeptide. The immunity is conferred by two different systems, the lipoprotein LanI and the ABC transporter LanFEG, protecting the host from the antimicrobial action of the lanthipeptide. The two-component regulatory system LanRK is initiated by extracellular active lanthipeptide and thereby activate the promoters (P*). (B) Comparative analysis of precursor nisin (NisA) and precursor subtilin (SpaS). The conserved FD/NLD box in the leader peptide are highlighted by the yellow box. A to E represent different rings. Ser and Thr that are involved in ring formation are shown in red. Ser and Thr that are dehydrated but not involved in ring formation are shown in green and purple. Cys that is involved in ring formation are indicated in blue. *, identical amino acid residues in NisA and SpaS. Download FIG S1, TIF file, 2.9 MB. Copyright © 2021 Chen et al. 2021 Chen et al. https://creativecommons.org/licenses/by/4.0/ This content is distributed under the terms of the Creative Commons Attribution 4.0 International license.

In 2020, we, for the first time, described the subcellular localization and assembly process of the nisin biosynthesis machinery in L. lactis and proposed a model for the assembly of the complex NisBTC, which is mainly located at the old cell poles (26). However, NisBTC clusters could only be visualized when nisin secretion was blocked by a point mutation in the ABC transporter NisT. Previous studies indicate that the enzymes and transporter could perform their respective functions independently of the other proteins. For example, the *in vitro* activity of NisB was demonstrated in the absence of NisC and NisT (27). Also, the *in vitro* reconstitution of the cyclization process mediated by NisC alone was reported (10). While NisT could transport unmodified or dehydrated precursor nisin in the absence of either NisB or NisC, the yield of the secreted peptide was severely decreased (28). It seems that the formation of the lantibiotic synthetase complex is not a prerequisite for the correct functioning of any of the enzymes but is crucial for the efficiency of peptide transportation, and the complex is probably highly unstable and transient in nature (2). This is likely to be the main reason for the difficulty in visually probing the assembly of the machinery by fluorescence microscopy and direct isolation of the whole complex from a wild-type situation in L. lactis. The cells of L. lactis are coccoid with a typical length of 0.5 to 1.5 μm. The cells of B. subtilis, a rod-shaped Gram-positive bacterium, are about 4.0 to 10.0 μm long, with a much larger cell volume than L. lactis, which is helpful for analysis of spatial distribution of proteins, DNA, RNA, lipids, and other biological macromolecules within the cells. In fact, the subcellular localization and cellular dynamics of a substantial number of proteins and protein complexes, such as the Sec machinery (29, 30), the Tat translocases (31), cell division proteins (32, 33), competence proteins, Clp proteases (34), penicillin-binding proteins (35, 36), sporulation proteins (37, 38), and the envelope stress-inducible two-component system LiaRS (39), have been investigated in B. subtilis. Moreover, B. subtilis ATCC 6633 is an efficient and natural producer of subtilin, a highly homologous lantibiotic to nisin (Fig. S1B and Table S1) (15, 40). Recently, subtilin production systems have been created in B. subtilis 168, WB800, and PG10 (40). We thus attempted to use B. subtilis as a host to investigate the subcellular localization of the nisin biosynthesis-associated components and gain a more detailed insight into the assembly process of the biosynthesis machinery. As numerous proteins and protein complexes often exhibit different localizations in coccoid- and rod-shaped cells, there are some main questions to answer. Will the enzymes and the transporter assemble into a complex in B. subtilis? If yes, will the subcellular distribution of the complex be similar to that in L. lactis? Is it assembled at the cell poles or not in B. subtilis? Is the assembly of the complex a rather static or dynamic process?

10.1128/mBio.01219-21.6 TABLE S1 Similarity and identity between the LanA, LanB, LanC, and LanT proteins of the nisin and subtilin systems. Download Table S1, DOCX file, 0.01 MB. Copyright © 2021 Chen et al. 2021 Chen et al. https://creativecommons.org/licenses/by/4.0/ This content is distributed under the terms of the Creative Commons Attribution 4.0 International license.

In this study, we report the direct visualization and the dynamic assembly of the functional nisin biosynthesis machinery in live bacteria by advanced fluorescence microscopy. At the single-cell level, NisBTC complexes were found to be assembled at specific regions throughout the cytoplasmic membrane. By probing the dynamic behavior of the modification machinery and the transporter, we gained in-depth insights into the assembly mechanism of the intact biosynthesis machinery, with the NBD domain of NisT as the membrane anchor for NisBC. Importantly, this study provides direct evidence for the existence of the lantibiotic biosynthesis machinery at the cell membrane and sheds light on the detailed association of its components.

## RESULTS

### Establishment of an effective production and secretion platform of fully modified precursor nisin in B. subtilis.

In our initial attempt to produce precursor nisin (NisA) in B. subtilis, the nisin biosynthetic operon *nisABTC* controlled by the nisin-inducible promoter (P*_nisA_*) was integrated into the chromosome of B. subtilis in which the two-component regulatory system NisRK had been introduced. However, the production of fully modified NisA was not detected after the induction by nisin Z, which was probably caused by the deficient expression of one or more proteins due to the wild-type ribosomal binding sites (RBSs) used, which were from L. lactis.

**FIG 1:**
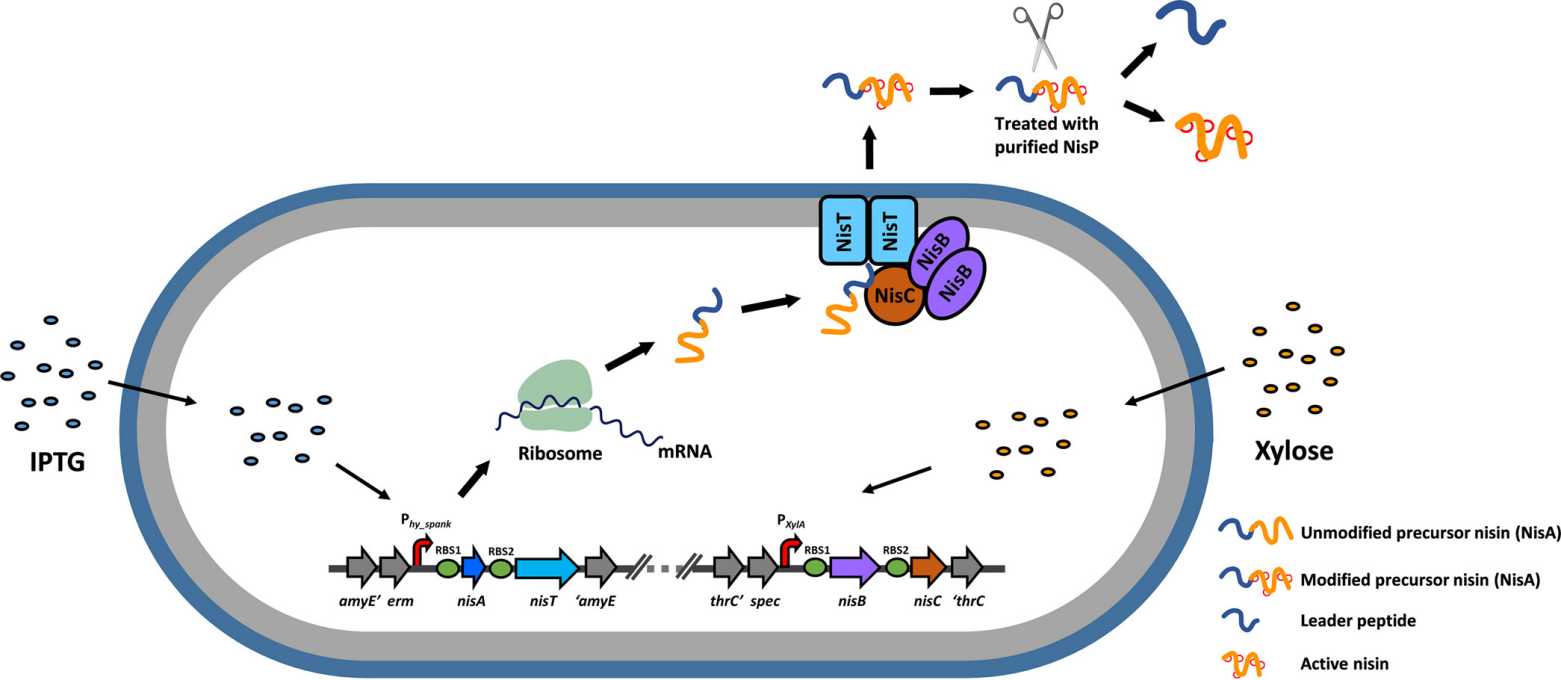
Overview of the heterologous production platform for precursor nisin in B. subtilis. Nisin biosynthesis-associated genes were integrated into the chromosome of B. subtilis via double-crossover reaction. The genes *nisAT* under the control of the IPTG-inducible promoter P*_hy_spanK_* are located in the *amyE* locus. The genes *nisBC* controlled by the xylose-inducible promoter P*_xylA_* are located in the *thrC* locus. RBS1 and RBS2, well-functional RBSs in B. subtilis (see Text S1). NisA, precursor nisin with a leader peptide and a core peptide. NisB, dehydratase which converts serines and threonine residues into dehydroalanine and dehydrobutyrine, respectively. NisC, cyclase which catalyzes the addition of a thiol group in cysteine to an N-terminally located dehydroamino acid, resulting in lanthionine rings. NisT, ABC transporter which transports fully modified NisA. With the induction by extracellular addition of IPTG and xylose, NisA, NisB, NisC, and NisT are expressed in the cells. NisA is modified by NisB and NisC and subsequently is exported by NisT. To activate the antimicrobial ability, the leader peptide could be removed using the *in vitro* cleavage by the protease NisP. NisB, NisC, and NisT presumably assemble a nisin biosynthetic and transportation machinery within the cytoplasmic membrane.

**FIG 2:**
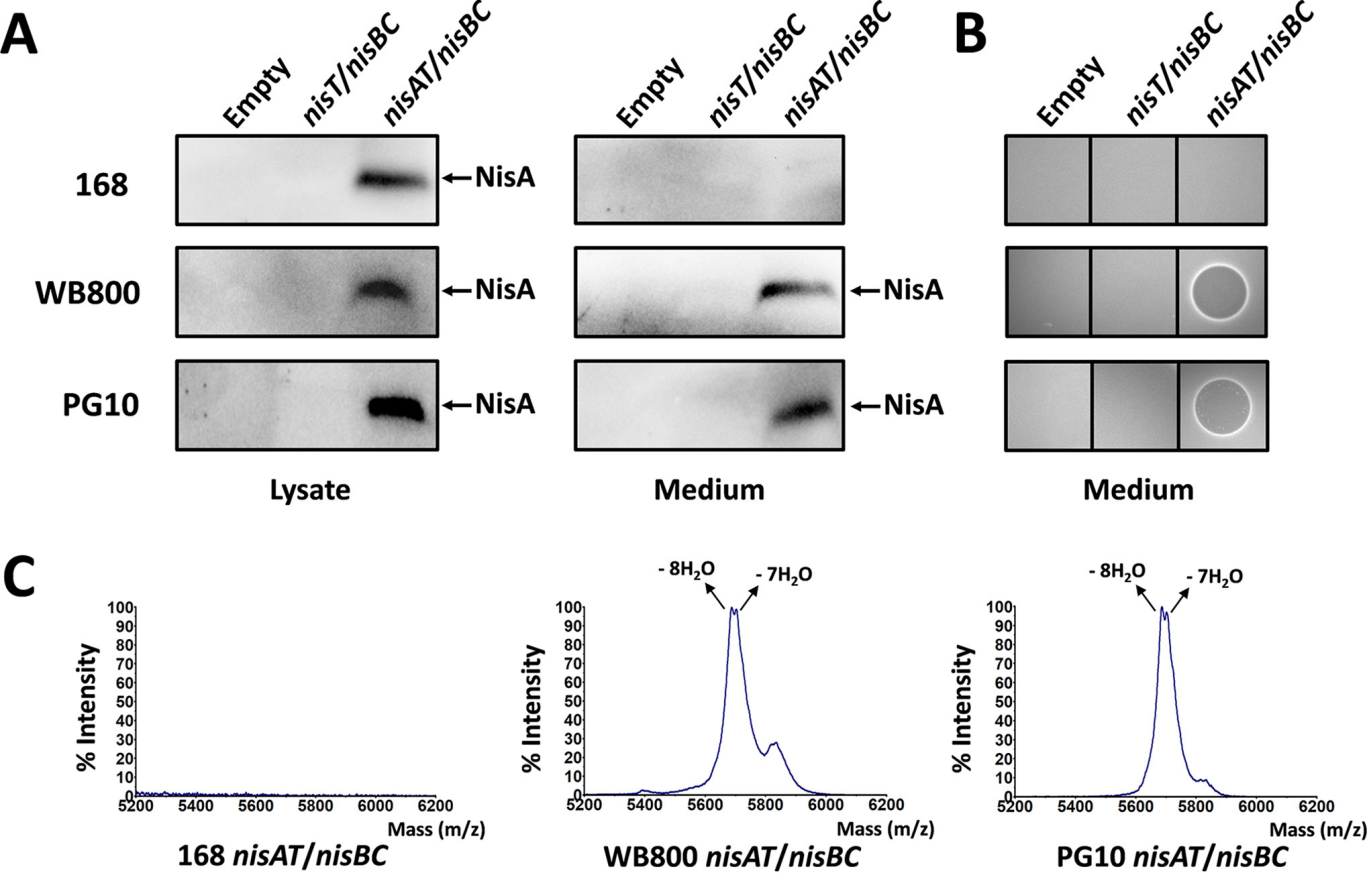
Heterologous production of precursor nisin by B. subtilis 168, WB800, and PG10. (A) Intracellular and extracellular precursor nisin (NisA) detected by Western blotting using antileader peptide antibody. For extracellular determination, the supernatant of cell culture was TCA precipitated. Empty, no integration of genes. (B) Antimicrobial activity assay of secreted NisA after removing leader peptide. The TCA-precipitated supernatant was incubated with the purified protease NisP at 30°C. The indicator strain is Micrococcus flavus. (C) MALDI-TOF MS data for TCA-precipitated supernatant.

To address this problem, we introduced the structural nisin gene *nisA* and the ABC transporter NisT-encoding gene under the control of an IPTG (isopropyl-β-d-thiogalactopyranoside)-inducible hyperspank promoter (P*_hy_spank_*) in the *amyE* locus and incorporated the transcriptional unit *nisBC* regulated by a slightly weaker and xylose-inducible promoter (P*_xylA_*) in the *thrC* locus. Importantly, the RBS sequences in front of all the genes were replaced by the well-functional RBSs in B. subtilis (Fig. 1). By using these two inducible promoters, we are able to resemble the relative higher expression level of *nisA* than *nisBC* while also circumventing the need for introduction and optimization of the nisin-controlled gene expression system (NICE). B. subtilis strains 168 and WB800 (41) and mini*Bacillus* strain PG10, of which the genome has been reduced by 36% (40, 42), were evaluated as production hosts. Mid-to-late exponentially growing cultures were induced with 0.5% (wt/vol) xylose and 0.1 mM IPTG simultaneously. Intracellular expression of NisA in all three strains was confirmed by Western blotting. After trichloroacetic acid (TCA) precipitation of the culture supernatant, the secreted NisA was detected for B. subtilis WB800 and PG10 with similar yield, but not for B. subtilis 168 (Fig. 2A). Moreover, after the *in vitro* removal of leader peptide by protease NisP, similar antimicrobial activity of both TCA-precipitated peptides was observed (Fig. 2B). In agreement with this, matrix-assisted laser desorption ionization–time of flight mass spectrometry (MALDI-TOF MS) indicated that the secreted NisA by B. subtilis WB800 and PG10 was fully dehydrated (Fig. 2C). The reason for the absence of NisA in the supernatant of B. subtilis 168 is likely the degradation of NisA by extracellular proteases (partial or full leader peptide cleavage) because both WB800 and PG10 lack all the five extracellular serine proteases of which at least AprE, WprA, and Vpr have been suggested to be involved in cleavage/degradation of precursor subtilin, a homologous lantibiotic of nisin (Table S1) (43).

10.1128/mBio.01219-21.10 TEXT S1 Materials and methods. Download Text S1, DOCX file, 0.02 MB. Copyright © 2021 Chen et al. 2021 Chen et al. https://creativecommons.org/licenses/by/4.0/ This content is distributed under the terms of the Creative Commons Attribution 4.0 International license.

In conclusion, we established a platform for effective production and secretion of the fully modified precursor peptide of nisin in a form without the removal of leader peptide in B. subtilis WB800 and PG10 (Fig. 1), which alleviates the need for the presence of immunity proteins. As extensive genome reduction leads to strong interference in gene regulation and metabolism and also causes marked changes in the growth and physiology of the bacteria (42), WB800 is more suitable than PG10 for the following study of the subcellular localization of nisin biosynthesis-related proteins.

### NisB, NisC, and NisT are all distributed in a punctate pattern along the cell periphery.

In order to track the subcellular distribution of the components associated with the putative nisin biosynthetic machinery, sfGFP (superfolder green fluorescent protein) and mKate2 (red fluorescent protein) were employed in this study (44). sfGFP and mKate2 were fused to the C terminus of the peptide NisA and the N and/or C termini of the enzymes NisB and NisC and the ABC transporter NisT, respectively. A Gly-rich chain was used as a flexible linker to fuse the nisin-related proteins with fluorescent proteins. This enables the modification of the C-terminal core peptide of NisA and keeps the modification and transport machinery to be functional when fused (45, 46). For convenience, the resulting strains are referred to according to the nisin biosynthesis-associated proteins that they produce, such as with “A_sfGFP_” being the NisA-sfGFP fusion or “B_mKate2_” being the NisB-mKate2 fusion. Thus, “A_sfGFP_-T/BC” represents a strain lacking the native peptide NisA but expressing the NisA-sfGFP fusion as well as NisB, NisT, and NisC. Among the constructions, sfGFP-NisB, mKate2-NisB, sfGFP-NisC, and sfGFP-NisT displayed a weak or almost no fluorescent signal. NisA-mKate2, NisC-mKate2, and NisT-mKate2 were found to be degraded largely in the cells. Eventually, we screened out NisA-sfGFP, NisB-sfGFP, NisB-mKate2, NisC-sfGFP, mKate2-NisC, NisT-sfGFP, and mKate2-NisT, which were stable and functional (giving rise to all combinations to correctly modify and transport NisA) and exhibited good signals in cells, enabling the following subcellular localization studies.

**FIG 3:**
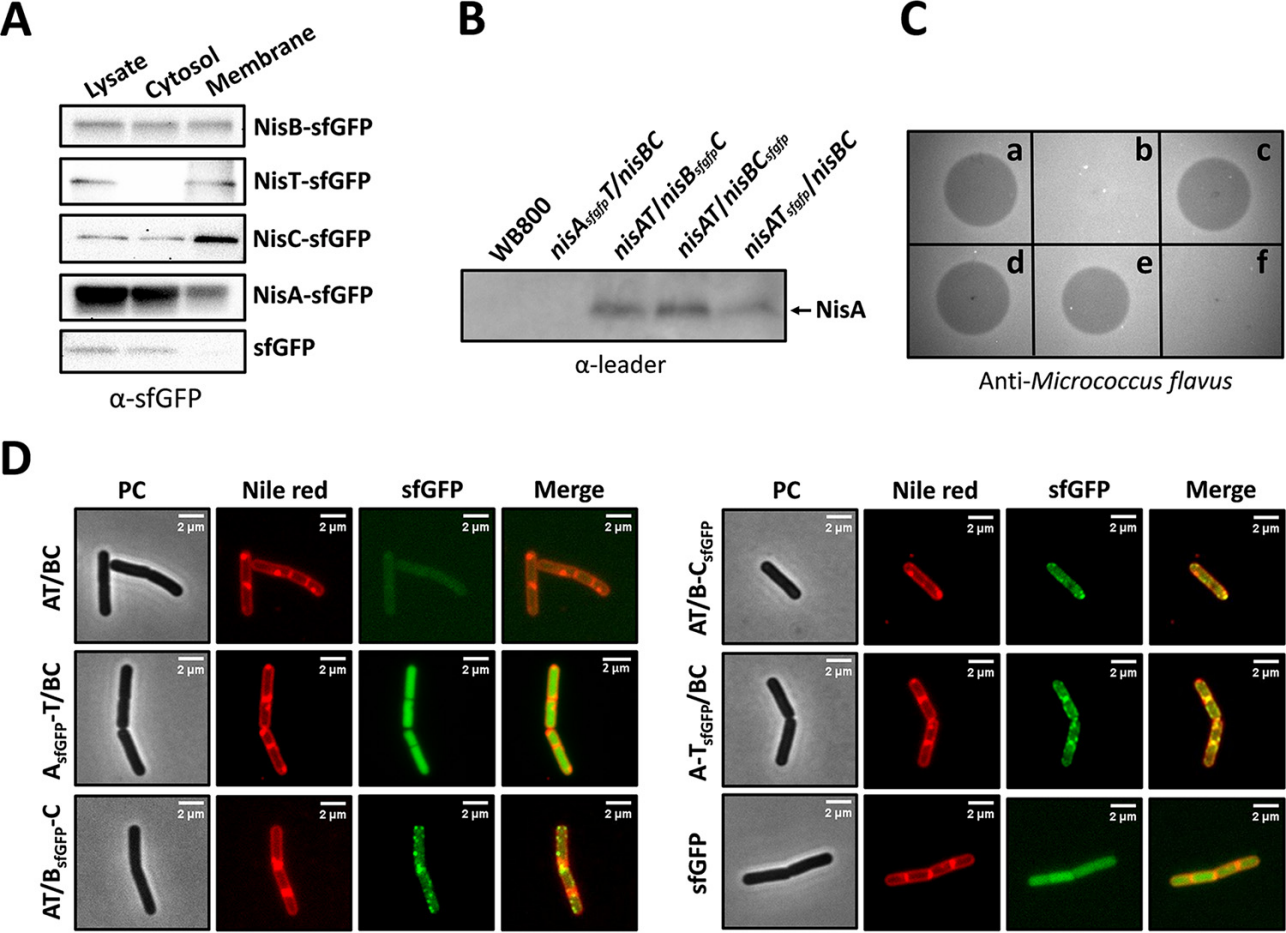
Determination of the subcellular localization of the nisin biosynthesis-associated proteins in B. subtilis WB800 using advanced fluorescence microscopy. (A) Western blotting of fusion proteins in the lysate, cytosol, and membrane fractions. NisB-sfGFP, NisT-sfGFP, NisC-sfGFP, and NisA-sfGFP were determined in different fractions of the strains AT/B_sfGFP_-C, A-T_sfGFP_/BC, AT/B-C_sfGFP_, and A_sfGFP_-T/BC, respectively. The strain WB800 *thrC*::P*_xylA_*-*sfgfp* is regarded as a control. The monoclonal anti-GFP antibody was used. (B) Detection of secreted NisA in TCA-precipitated supernatant by Western blotting. WB800 is employed as a negative control. The antileader peptide antibody is used. (C) Antimicrobial activity assay. The samples are a TCA-precipitated culture supernatant, which was incubated with the purified protease NisP at 30°C to remove the leader peptide *in vitro*. (a) Strain AT/BC. (b) Strain A_sfGFP_-T/BC. (c) Strain AT/B_sfGFP_-C. (d) Strain AT/B-C_sfGFP_. (e) Strain A-T_sfGFP_/BC. (f) Strain WB800 containing single *sfgfp* gene. The indicator strain is M. flavus. (D) Subcellular distribution of NisA-sfGFP, NisB-sfGFP, NisC-sfGFP, and NisT-sfGFP in WB800 with the intact nisin biosynthesis machinery. Cells were stained by Nile red to shown the membrane localization. PC, phase contrast.

Western blotting showed that NisA-sfGFP, NisB-sfGFP, NisC-sfGFP, and NisT-sfGFP were expressed in the cells. To evaluate whether fusion of one of the proteins to sfGFP affects the protein localization, the cell fractions of cytosol and membrane were separated (Fig. 3A). As expected, NisT-sfGFP was mainly detected in the membrane, and the single sfGFP was only observed in the cytosol. NisA-sfGFP, NisB-sfGFP, and NisC-sfGFP were found to be present in both cytosol and membrane in accordance with their localization determined in L. lactis (26). According to the proposed nisin biosynthesis machinery in L. lactis (14), the existence of all the four fusion proteins in the membrane fraction implied that NisA, NisB, NisC, and NisT probably also form a membrane-associated complex in B. subtilis. It has been shown in a previous study that fusing sfGFP to the C terminus of NisA neither prevents the binding of NisA to NisB and NisC nor affects the modification of the core peptide, whereas NisA-sfGFP could not be secreted, likely due to the large attachment of sfGFP to NisA (26). The ability of NisB-sfGFP, NisC-sfGFP, and NisT-sfGFP to modify or transport NisA was assessed. In these three cases, secreted NisA was detected with a similar yield in the culture supernatant (Fig. 3B) and displayed very similar antimicrobial activity after *in vitro* cleavage of the leader peptide by NisP (Fig. 3C). This indicated that the functionality of the fusion proteins was almost indistinguishable from that of the wild-type enzymes or transporter. Overall, we conclude that the sfGFP-labeled components of the nisin modification and transport machinery could well be employed to study the subcellular distribution of wild-type proteins.

Cells from the midexponential growth phase were examined by fluorescence microscopy (Fig. 3D). Strikingly, the fluorescence imaging of living cells revealed a homogeneous distribution of NisA-sfGFP throughout the cells of the strain A_sfGFP_-T/BC. In contrast, NisB-sfGFP and NisC-sfGFP were found to be concentrated into relatively intense foci at the cell poles, the septum, and intermediate positions along the periphery of growing cells of AT/B_sfGFP_-C and AT/B-C_sfGFP_, respectively, with diffuse cytoplasmic fluorescence also visible in most cells, consistent with the detection of the corresponding proteins in the cytosol, as described above. In the strain A-T_sfGFP_/BC, NisT-sfGFP was distributed in a mixed pattern of foci and “patchy” fluorescence tending to the cell periphery. By estimating the spatial location of the visible foci with reference to the Nile red-stained membrane, we found that the fluorescence foci of NisB-sfGFP, NisC-sfGFP, and NisT-sfGFP mostly appeared close to/in the cell membrane. The localization pattern of fusion proteins was further verified by comparable studies in which NisB, NisC, and NisT were tagged by mKate2 (Fig. S2A). Similar to the sfGFP fusions, NisB-mKate2, mKate2-NisC, and mKate2-NisT were fully functional, as was confirmed by the antimicrobial activity assay (Fig. S2B). The fluorescence signal from sfGFP or mKate2 expressed alone was always homogeneously distributed in the cytoplasm of B. subtilis cells (Fig. 3D and Fig. S2A). Taken together, we observed that the enzymes NisB and NisC, together with the ABC transporter NisT, are distributed in a punctate pattern along the cell periphery.

10.1128/mBio.01219-21.2 FIG S2 Subcellular localization of NisB, NisC, and NisT in B. subtilis WB800 with the intact nisin biosynthesis machinery. (A) Punctate localization of NisB-mKate2, mKate2-NisC, and mKate2-NisT in the presence of other components. B. subtilis WB800 carrying single *mKate2* is used as a control. (B) Antimicrobial activity assay. TCA-precipitated supernatant was incubated with the purified NisP at 30°C to remove the leader peptide *in vitro*. (1) Strain AT/BC. (2) Strain AT/B_mKate2_-C. (3) Strain AT/B-_mKate2_C. (4) Strain A-_mKate2_T/BC. The indicator strain is M. flavus. Download FIG S2, TIF file, 1.8 MB. Copyright © 2021 Chen et al. 2021 Chen et al. https://creativecommons.org/licenses/by/4.0/ This content is distributed under the terms of the Creative Commons Attribution 4.0 International license.

### Direct visualization of the functional nisin biosynthesis machinery NisBTC.

Although it has been shown that the nisin biosynthesis machinery NisBTC with a mutation in the H-loop of the NBD domain of NisT is assembled and clearly observed at the old cell poles in L. lactis (26), the wild-type complex NisBTC has neither been isolated successfully nor visualized directly. We therefore aimed at capturing the assembly of such a complex by using different fluorescent proteins to label nisin-related proteins in B. subtilis.

**FIG 4:**
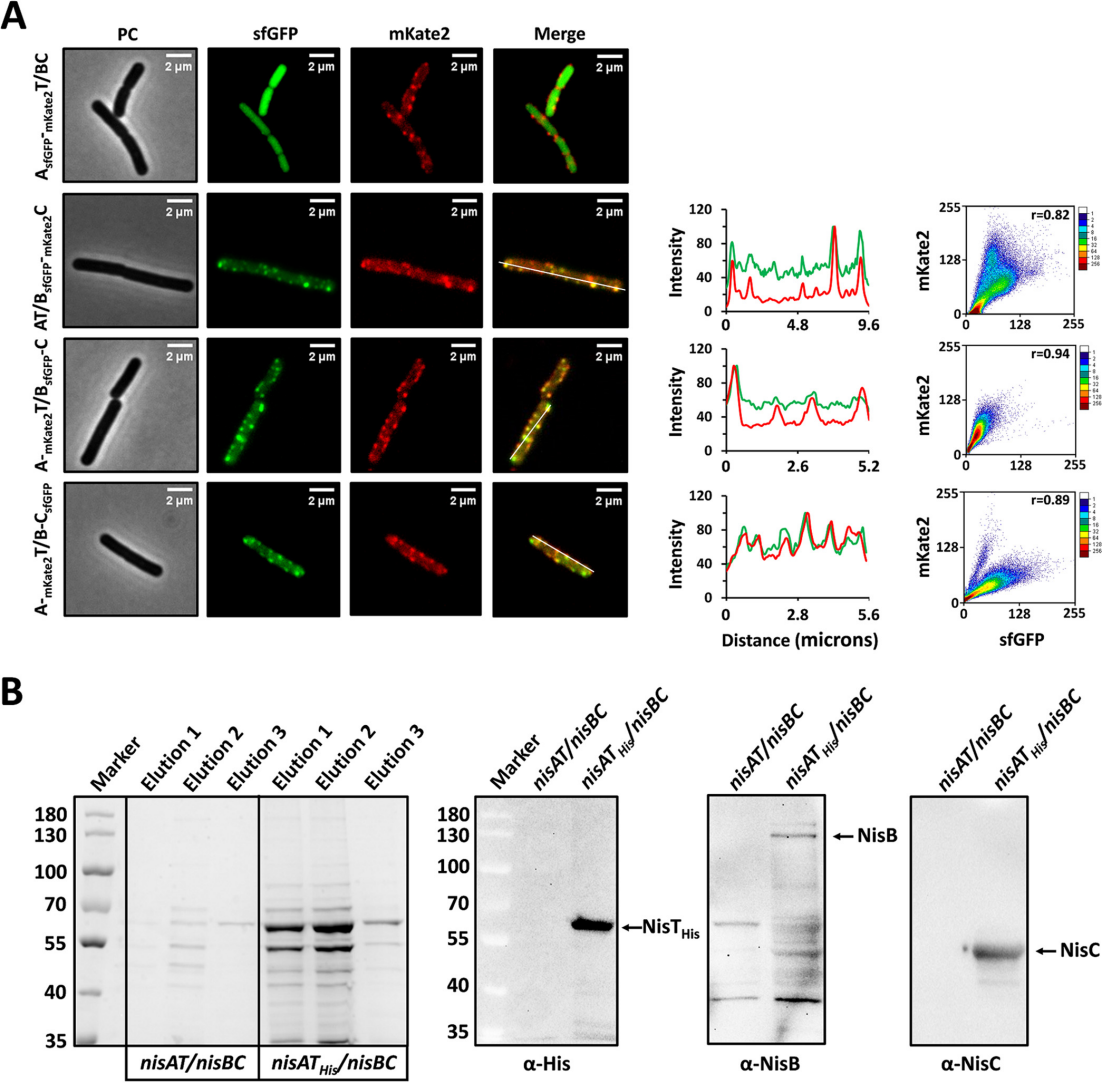
Direct visualization of the complex NisBTC foci associated with the cytoplasm membrane. (A) Colocalization of NisB, NisC, and NisT. NisB-sfGFP and mKate2-NisC, mKate2-NisT and NisB-sfGFP, and mKate2-NisT and NisC-sfGFP were colocalized at the same spots within the cell membrane in corresponding strains (fluorescence images on the left). Fluorescence profile by linescan is shown in linear graph (in the middle). The analysis is based on the yellow line in the merged images. Green curve, sfGFP signal. Red curve, mKate2 signal. Pearson’s correlation coefficient (PCC; *r*) between the green foci and red foci is shown in scatter diagram (right). The PCC has range of +1 (perfect correlation) to −1 (perfect but negative correlation), with 0 denoting the absence of a relationship. (B) Pulldown assay with His-tagged NisT. The membrane protein NisT_His_ was purified by Ni-NTA purification from the membrane fraction. Elutions (1, 2, and 3) were applied in 8% SDS-PAGE. Elution 2 was analyzed by Western blotting using anti-His, anti-NisB, and anti-NisC antibodies. Marker, the protein ladder. NisT_His_ size, 70 kDa. NisB size, 117.5 kDa. NisC size, 47.9 kDa.

In the strain A_sfGFP_-_mKate2_T/BC, NisA-sfGFP and mKate2-NisT displayed apparently different subcellular distributions. Due to fast and abundant expression of precursor nisin, it is difficult to probe the interaction between NisA and its transporter NisT. Nevertheless, regarding NisA-sfGFP as background, the multiple export sites for the substrate, i.e., the NisT foci, around the cells were clearly exhibited. When NisB-sfGFP and mKate2-NisC were coexpressed in the presence of NisA and NisT, their clusters were always colocalized, which was further verified by the comparison of fluorescence intensity profiles within the cells and the quantitative colocalization analysis (Pearson’s correlation coefficient, *r* = 0.82). The colocalization of NisB with NisC was in line with the *in vivo* isolation and *in vitro* assembly of the nisin modification machinery (16, 17). Similarly, mKate2-NisT was coexpressed with either NisB-sfGFP or NisC-sfGFP, while other components of the nisin biosynthesis machinery were present. Significantly, the fluorescence images and the quantitative analyses indicated that the mKate2-NisT foci colocalized with the foci of both NisB-sfGFP and NisC-sfGFP (Fig. 4A). These above data demonstrate that simultaneous colocalization of all three proteins occurs at discrete spots distributed across the membrane, being visualized here directly. This distribution pattern was reinforced by the observation of the same cellular localization of NisBTC foci in another B. subtilis host, i.e., PG10 (Fig. S3). To offer further evidence for the existence of NisB, NisC, and NisT in the cell membrane of B. subtilis, functioning as a complex NisBTC, the pulldown assay with the purification of NisT was conducted (Fig. 4B). A 6×His tag was fused to the C terminus of NisT, mediated by a factor Xa cleavage sequence. Elution fractions from the strain *nisAT_His_*/*nisBC* were applied in SDS-PAGE, and an intense band of the size corresponding to NisT (∼69 kDa) was observed. Western blotting showed that both NisB and NisC were copurified, thus directly revealing the fact that NisB, NisC, and NisT were assembled into the NisBTC complex within the cell membrane.

10.1128/mBio.01219-21.3 FIG S3 Subcellular localization of NisT in B. subtilis PG10 with the functional nisin biosynthesis machinery. In the upper lane, sfGFP was fused to the C terminus of NisT. In the lower lane, mKate2 was fused to the N terminus of NisT. Download FIG S3, TIF file, 2.1 MB. Copyright © 2021 Chen et al. 2021 Chen et al. https://creativecommons.org/licenses/by/4.0/ This content is distributed under the terms of the Creative Commons Attribution 4.0 International license.

Based on the above results, we conclude that the direct visualization of the wild-type complex NisBTC was successfully carried out and the complex was distributed in discrete foci circumferentially surrounding the cells. To our knowledge, this is the first study to provide direct evidence for the existence of the complex NisBTC by presenting its subcellular distribution.

### NisBC from the cell pole or septum is targeted to NisT localized in the membrane to initiate the assembly of NisBTC.

We have probed the *in vivo* formation of NisBTC patches in the cytoplasmic membrane, but the details concerning the assembly process of these foci are still unclear. Does NisT play a role as the membrane anchor for NisB and NisC? During the assembly of NisBTC, what is the corresponding movement route for the proteins within the cells? When NisT is deficient, will NisB and NisC still be targeted to specific locations within the membrane? If not, what position in bacterial cells are they localized at? To address these questions, we employed the strategy of timed expression of target proteins to examine the dynamic behavior of the machinery assembly.

**FIG 5:**
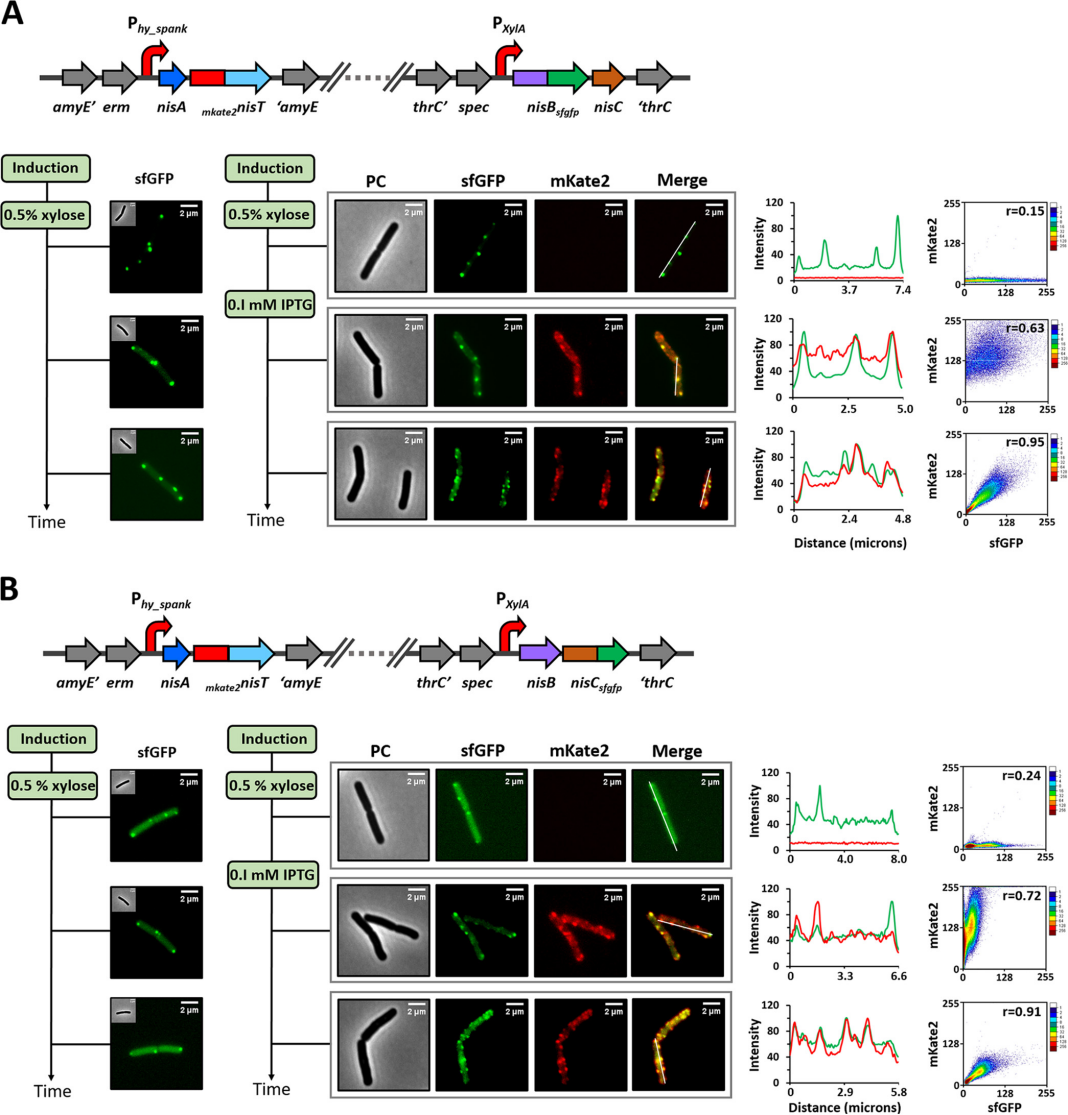
Demonstration of the dynamic assembly of the nisin biosynthesis machinery NisBTC by time-resolved expression strategy. (A) NisB-sfGFP initially was confined to the cell poles and septum and was increasingly colocalized with mKate-NisT within the cell membrane with time. (B) NisC-sfGFP originally was restricted to the cell poles and septum and was increasingly colocalized with mKate-NisT within the cell membrane with proceeding time. In panels A and B, 0.5% (wt/vol) xylose was added as inducer at 0 min, followed by the induction by 0.1 mM IPTG at 30 min. In the control, just 0.5% (wt/vol) xylose was added as inducer throughout the growth. PC, phase contrast. Fluorescence profile by linescan is shown in linear graph. The analysis is based on the yellow line in the merged images. Green curve, sfGFP signal. Red curve, mKate2 signal. Pearson’s correlation coefficient (PCC; *r*) between the green foci and red foci is shown in scatter diagram. The PCC has range of +1 (perfect correlation) to −1 (perfect but negative correlation), with 0 denoting the absence of a relationship.

In the strain A-_mKate2_T/B_sfGFP_-C, the artificial operon *nisA-_mKate2_nisT* was regulated by the promoter P*_hy_spank_*, and *nisB_sfgfp_-nisC* was under the control of the promoter P*_xylA_*. Thus, the expression order of the enzymes and transporter could be adjusted by adding the appropriate inducer at different growth phases. Initially (0 min), xylose was added into the medium to a final concentration of 0.5% (wt/vol) at the midexponential growth phase. After a while (30 min), fluorescence microscopy strikingly demonstrated that NisB-sfGFP was almost exclusively localized at the cell poles and septum. The fluorescence signal of mKate2-NisT was not detected, as expected, due to the lack of inducer. Subsequently, 0.1 mM IPTG was added to the cell culture. In a while (60 min), later-expressed mKate2-NisT became visible and was found to be distributed in discrete foci located throughout the cytoplasmic membrane. In the meantime, NisB-sfGFP began to appear enriched around partial NisT foci in the membrane, next to the cell poles and septum, which was regarded as the process of a primary assembly of NisBTC. Finally (90 min), we observed that nearly all the mKate2-NisT foci were well occupied by NisB-sfGFP foci. In parallel, the fluorescence intensity profiles within the cells gave a tendency for NisB-sfGFP and mKate2-NisT to be increasingly colocalized with time. Moreover, the increased Pearson’s correlation coefficient (from 0.63 to 0.95) after mKate2-NisT expression also supported the dynamic behavior of NisB-sfGFP being recruited to mKate2-NisT (Fig. 5A). In contrast, when xylose was used as sole inducer, NisB-sfGFP was exclusively localized at the cell poles and septum throughout the whole growth. As reported in previous studies, NisC is regarded to be associated with NisB, and they form a complex, thus functioning as a modification machinery (16, 17). We were wondering whether NisC also shows a similar behavior during the assembly process of NisABTC. As expected, in the strain A-_mKate2_T/B-C_sfGFP_, when only the production of NisB and NisC-sfGFP was induced, NisC-sfGFP was confined to the cell poles and septum, with diffuse cytoplasmic fluorescence also visible in most cells. As soon as NisA and mKate2-NisT were expressed, NisC-sfGFP started to leave from the cell poles and septum, and appeared nearby mKate2-NisT foci, until the dual fluorescent foci were highly colocalized. The dynamic behavior of NisC-sfGFP was further confirmed by the analysis of fluorescence intensity profiles and Pearson’s correlation method (Fig. 5B). In summary, we demonstrate the *in vivo* dynamic assembly of the nisin biosynthesis machinery NisABTC in rod-shaped cells, and, more importantly, the recruitment process of the NisABC complex from the cell poles and septum to NisT located in spots at the membrane was revealed.

### The substrate NisA is not necessary for the formation of the nisin biosynthesis-associated subcomplexes.

The data described above indicate the subcellular localization and the dynamic assembly of the intact nisin biosynthetic machinery. However, the subcellular distribution of the corresponding components in mutant backgrounds of the intact apparatus remains to be characterized.

**FIG 6:**
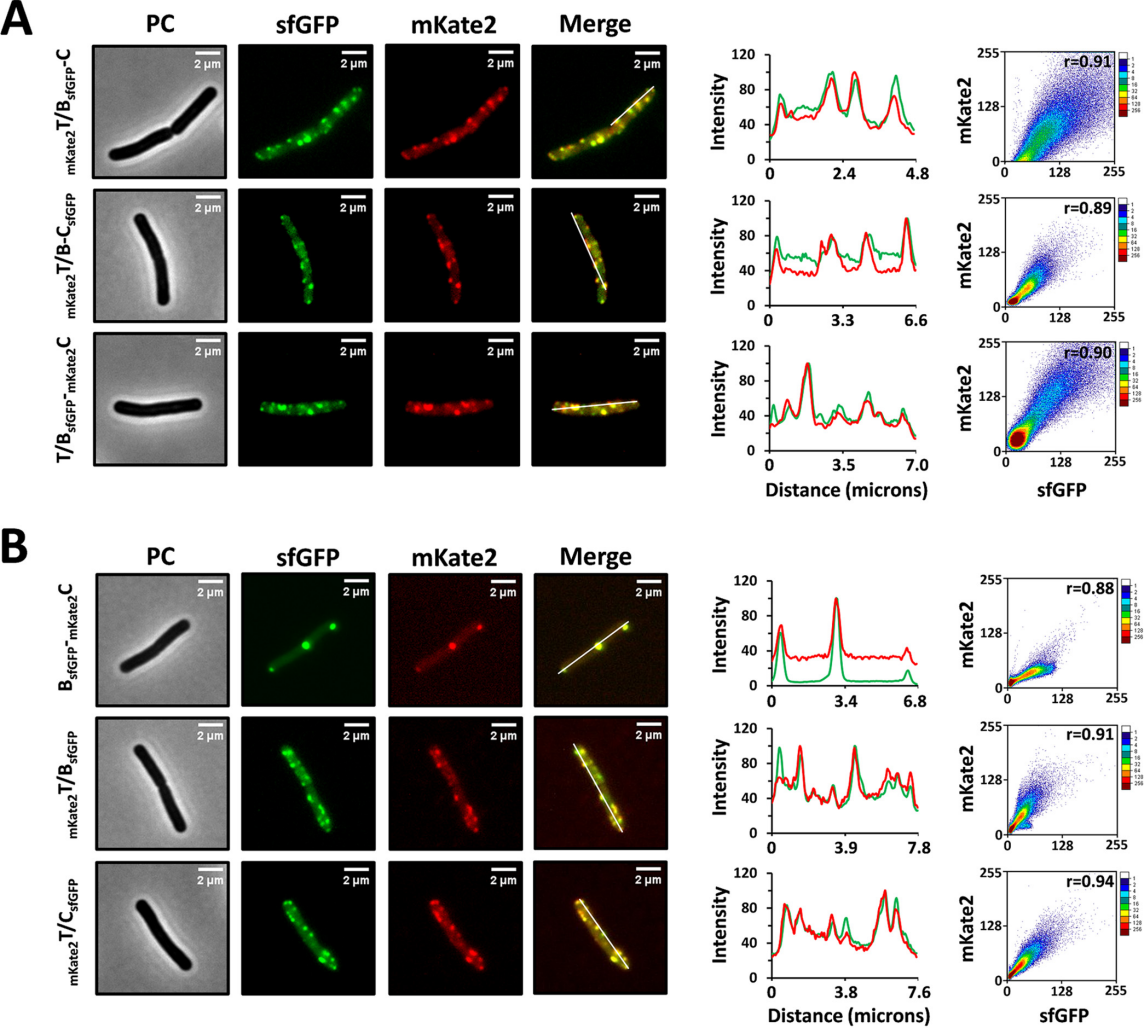
Assembly of the nisin biosynthesis-associated subcomplexes in B. subtilis WB800. (A) Colocalization of mKate2-NisT and NisB-sfGFP, mKate2-NisT and NisC-sfGFP, or NisB-sfGFP and mKate2-NisC at discrete spots along the cell membrane in the absence of NisA. (B) Colocalization of NisB-sfGFP and mKate2-NisC, mKate2-NisT and NisB-sfGFP, or mKate2-NisT and NisC-sfGFP in corresponding strains. PC, phase contrast.

Initially, we checked whether the substrate peptide is required for the assembly of NisBTC. Therefore, the dual fluorescently labeled strains _mKate2_T/B_sfGFP_-C, _mKate2_T/B-C_sfGFP_, and T/B_sfGFP_-_mKate2_C were constructed. sfGFP- and mKate2-tagged proteins appeared as independent clusters within the cell membrane, and they were further confirmed to be colocalized by the analysis of fluorescence images in all three cases (Fig. 6A). This result suggests that the assembly of NisBTC is not substrate dependent. To characterize the interactions between NisB, NisC, and NisT, different expression combinations with dual fluorescently labeling were generated (Fig. 6B). In the above timing expression experiment, when the expression of NisA and NisT was absent, NisB and NisC were concentrated at the cell poles and septum. Hence, as expected, in the strain B_sfGFP_-_mKate2_C, NisB-sfGFP was colocalized with mKate2-NisC at the regions of the cell poles and septum, in agreement with the polar localization of the modification machinery shown in L. lactis (26). In the strains _mKate2_T/B_sfGFP_ and _mKate2_T/C_sfGFP_, the foci of NisB-sfGFP and NisC-sfGFP were observed to be colocalized with mKate2-NisT foci, respectively, and their distribution is similar to that of the intact nisin biosynthesis machinery. These data revealed that NisB, NisC, and NisT were capable of associating with each other directly, implying that their association was not triggered by the presence of the substrate NisA. Previously, it was reported that NisA is necessary for the formation of NisBC, which was conducted *in vitro* (17). The conflicting results are possibly caused by different environments for complex formation. In summary, in the absence of the substrate, the subcomplexes that include NisBTC, NisBC, NisBT, and NisTC could still be formed. The recruitment of NisB, NisC, or NisBC to the cell membrane is specifically dependent on the transporter NisT.

### Homogeneously distributed NisT is recruited to the machinery by the modification complex NisBC during the assembly process.

Having revealed the subcellular localization of the enzymes and transporter in a series of conditions, we were curious about their distribution when they are expressed singly in the cells. The strains with the chromosomal integration of single *nisB_sfgfp_*, *nisC_sfgfp_*, and *nisT_sfgfp_* were made. We observed that the fluorescence signal of NisB-sfGFP and NisC-sfGFP were uniformly distributed in the cytoplasm, respectively, which was in line with their property of cellular proteins (Fig. S4A and B). Above, we have shown that with coexpression of NisT, the enzymes NisB and NisC were not diffusely located in the cytoplasm but localized in specific regions within the membrane, again emphasizing a recruiter role of NisT, to target NisB and NisC to the membrane. Interestingly, when only NisT-sfGFP was expressed in the cells, it was found to be homogeneously and circumferentially distributed in the cytoplasmic membrane without any enhanced bright foci (Fig. S4C). This finding was apparently distinguishable from the punctate distribution of NisT in the strain carrying the complete nisin biosynthetic gene cluster. Furthermore, as observed above, when both NisB and NisC or only NisB or NisC were coexpressed, NisT was not distributed uniformly but was confined to small clusters in the cell membrane. The difference in subcellular distribution between single NisT and NisT associated with NisB/NisC/NisBC implied a recruitment behavior of empty NisT homogeneously distributed in the cell membrane to the assembled NisBTC clusters by the modification machinery NisBC once the initial assembly of NisBTC is completed, promoting the growth of a strong nisin biosynthesis and transportation machinery. Similar recruitment behavior of NisT has been reported in L. lactis (26), with the difference that NisT that was diffusely present within the membrane was captured by NisBC mainly at a single spot, the old cell pole. In B. subtilis, we regard this process as the second step for the entire assembly.

10.1128/mBio.01219-21.4 FIG S4 Subcellular distribution of NisB, NisC, and NisT when they were expressed alone in B. subtilis WB800. (A) NisB-sfGFP was diffuse in the cytoplasm. (B) NisC-sfGFP was diffusely located in the cytoplasm. (C) NisT-sfGFP was uniformly distributed in the cytoplasmic membrane. Download FIG S4, TIF file, 2.4 MB. Copyright © 2021 Chen et al. 2021 Chen et al. https://creativecommons.org/licenses/by/4.0/ This content is distributed under the terms of the Creative Commons Attribution 4.0 International license.

### The NBD domain of NisT plays a role as a membrane anchor for NisBC beyond nucleotide binding and hydrolysis.

**FIG 7:**
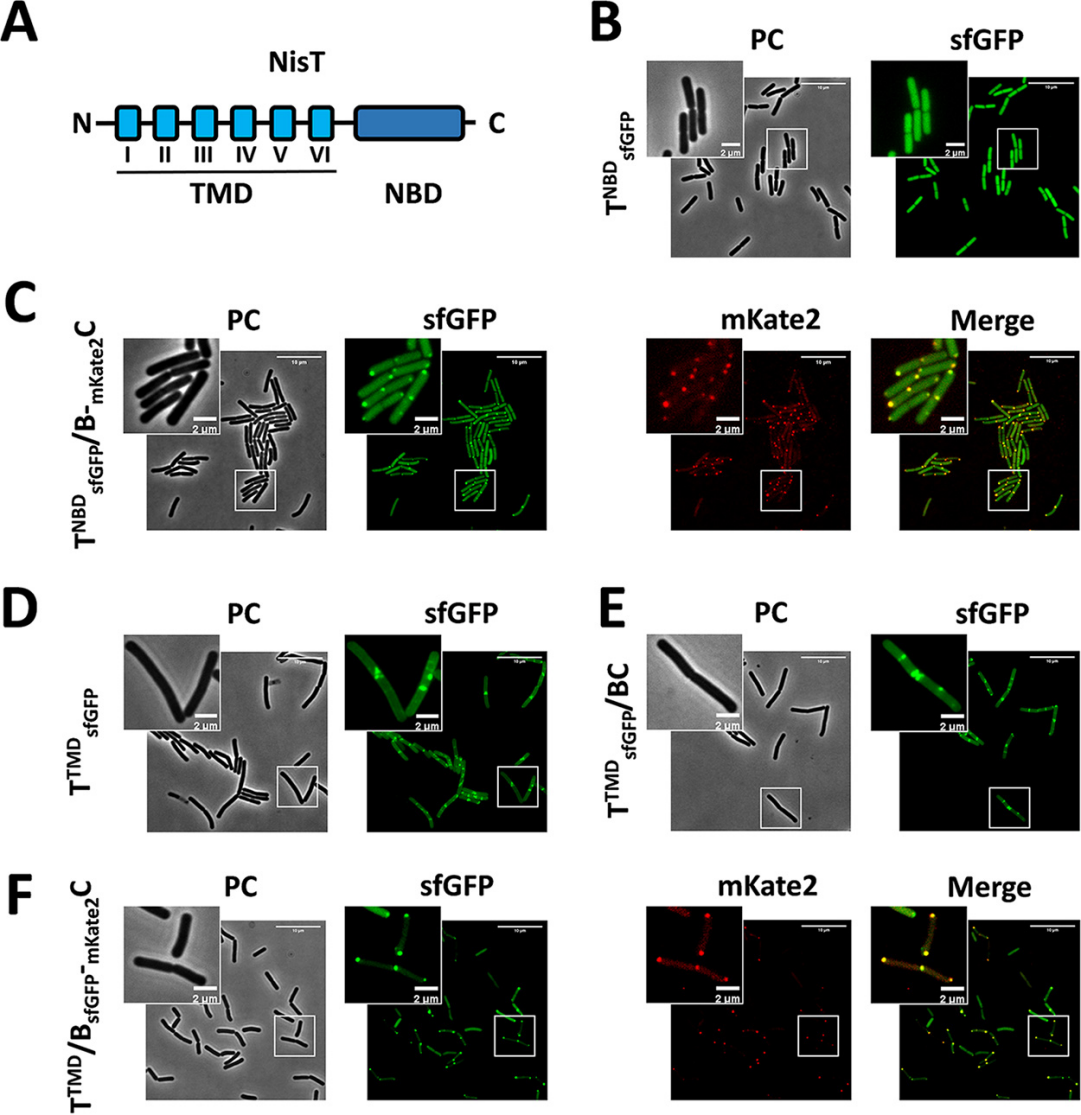
NisT interacts with NisBC via its NBD domain. (A) Domain organization of the ABC transporter NisT. NisT consists of a transmembrane domain (TMD) and a nucleotide binding domain (NBD). (B) Subcellular distribution of NisT^NBD^-sfGFP when it was expressed alone. (C) Colocalization of NisT^NBD^-sfGFP and mKate2-NisC at the cell poles and septum in the presence of NisB. (D) Subcellular distribution of NisT^TMD^-sfGFP when only it was expressed. (E) Subcellular distribution of NisT^TMD^-sfGFP with coexpression of NisB and NisC. (F) Colocalization of NisB-sfGFP and mKate-NisC at the cell poles and septum in the presence of NisT^TMD^. PC, phase contrast.

We have shown that NisBC was recruited to the membrane by NisT during the initial assembly of NisBTC. Therefore, there must be a molecular interaction between them. The ABC transporter NisT consists of a transmembrane domain (TMD) and a nucleotide binding domain (NBD) (Fig. 7A). We were wondering which domain is responsible for interacting with NisBC and therefore able to target it to the membrane. For this purpose, NisT was separated into its TMD and NBD domains, and both were C-terminally labeled by sfGFP. When NisT^NBD^-sfGFP was expressed alone, its distribution was diffuse within the cells as expected (Fig. 7B). In the strain T^NBD^_sfGFP_/B-_mKate2_C, NisT^NBD^-sfGFP was no longer diffuse but confined to the cell poles and septum in the majority of cells. Moreover, merged images indicated that NisT^NBD^-sfGFP was located in the spot where mKate2-NisC was present at the regions of the cell poles and septum (Fig. 7C). As fluorescently labeled NisC-associated foci represent the modification complex, we conclude that the polar bright foci are the assembled complex NisBT^NBD^C. These observations suggest that the modification machinery NisBC interacts with NisT^NBD^ and appears to be the driver of NisT^NBD^ to the cell poles and septum. When NisT^TMD^ was C-terminally tagged by sfGFP and was expressed singly, NisT^TMD^-sfGFP was circumferentially distributed in the membrane (Fig. 7D). The introduction of the coexpression of NisB and NisC did not result in any change in its subcellular localization (Fig. 7E). Furthermore, in the strain T^TMD^/B_sfGFP_-_mKate2_C, NisBC was not targeted to the membrane but was still restricted to the cell poles and septum (Fig. 7F). Compared to the distribution of NisBC foci associated with full-length NisT, we believe that NisBC lost the punctate distribution due to the deficiency of the NBD domain in NisT. Together, we conclude that the NBD domain of NisT functions as a membrane anchor for NisBC beyond nucleotide binding and hydrolysis.

In an attempt to identify the interaction sites between the NBD domain of NisT and NisB/NisC, the effect of mutagenesis of 11 highly conserved amino acids located in the NBD domain of NisT on the recruitment of NisB/NisC to the membrane was evaluated (Fig. S5). Mutations of residues G386, G389, K392, G408, I410, S496, Q499, Q501, R507, D519, and D526 to Ala were generated. However, in all the mutant cases, the enzymes NisB and NisC were still targeted to the membrane and concentrated in discrete foci along the cell periphery (Table S2). The distribution pattern was identical to that in the wild-type situation. This implies that the interaction between the transporter and enzymes is not restricted to certain single residues but is presumably mediated by more complex motifs and is not directly coupled to nucleotide binding or hydrolysis.

10.1128/mBio.01219-21.5 FIG S5 Sequence alignment of NisT with select other class I lanthibiotic transporters. The residues mutated in this study are boxed, with NisT residue numbers on top. Amino acids in yellow, transmembrane domain. Amino acids in blue, nucleotide binding domain (NBD). Amino acids in gray, similarity of sequence. Download FIG S5, TIF file, 2.2 MB. Copyright © 2021 Chen et al. 2021 Chen et al. https://creativecommons.org/licenses/by/4.0/ This content is distributed under the terms of the Creative Commons Attribution 4.0 International license.

10.1128/mBio.01219-21.7 TABLE S2 Subcellular localization of NisB and NisC when conserved residues of NisT are mutated. Download Table S2, DOCX file, 0.02 MB. Copyright © 2021 Chen et al. 2021 Chen et al. https://creativecommons.org/licenses/by/4.0/ This content is distributed under the terms of the Creative Commons Attribution 4.0 International license.

### NisBTC foci are static and potentially associated with lipid rafts.

**FIG 8:**
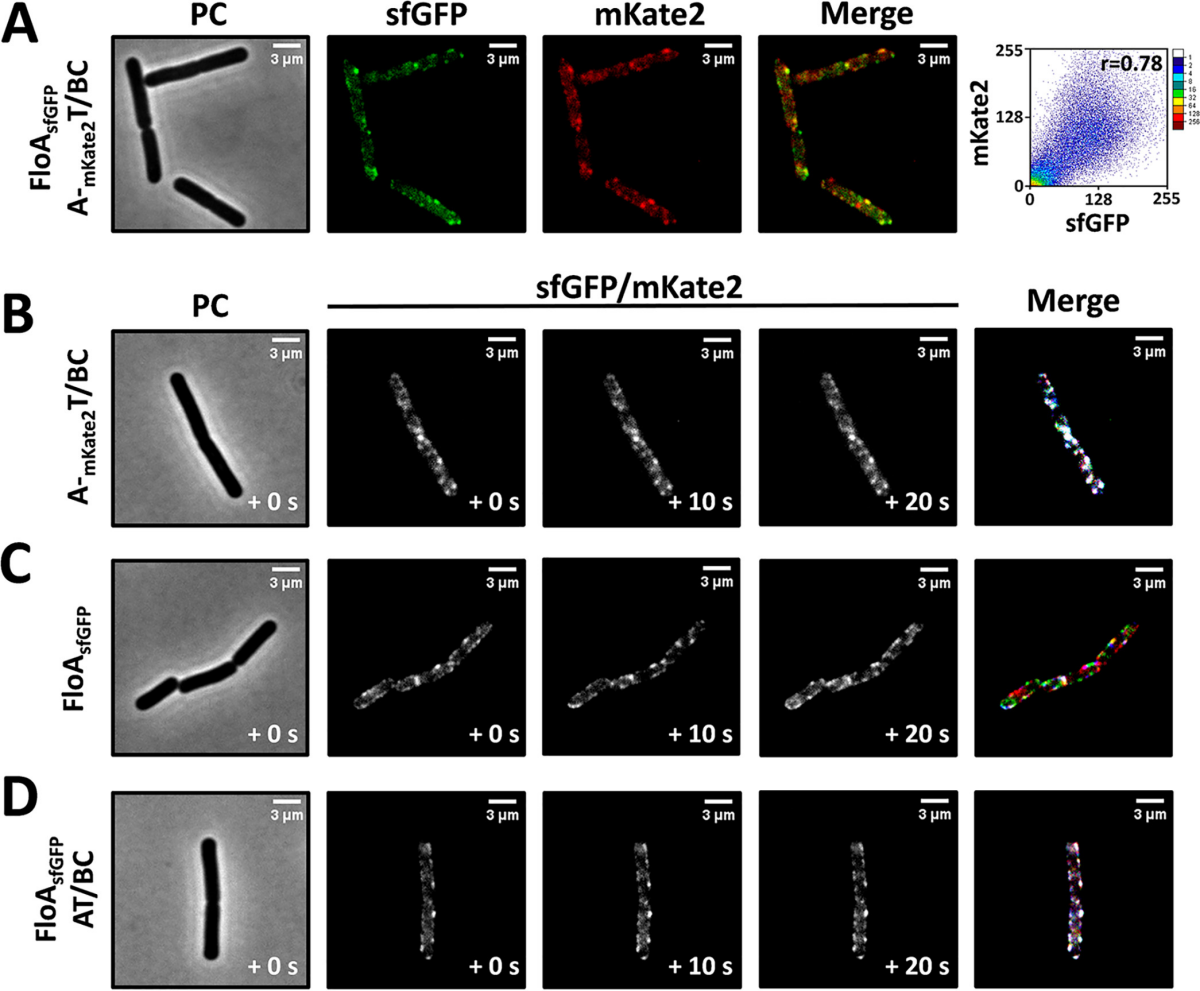
The nisin biosynthesis complex NisBTC is potentially associated with lipid rafts. (A) mKate2-labeled nisin biosynthetic machinery is colocalized with sfGFP-labeled FloA. FloA, a marker of lipid rafts in B. subtilis. (B to D) Representative sets of fluorescence images of mKate2-NisT (B) and FloA-sfGFP (C and D) acquired at 10-s intervals in corresponding strains. From left to right, phase contrast, three frames, and an overlay are shown. In the merged images of panels B to D, the signal from the three frames is colored consecutively red, green, and blue; any signal present in all three frames will appear white, whereas movement will result in the appearance of color.

Bacterial membranes have functional membrane microdomains (FMMs), a structure homologous to eukaryotic lipid rafts. Similar to their eukaryotic counterparts, bacterial FMMs harbor scaffold proteins termed flotillins that are thought to promote interactions between proteins spatially confined to the FMMs (47). Flotillins also play a role in maintaining membrane heterogeneity and regulating membrane fluidity. In B. subtilis, the FMMs contain two different flotillin-like proteins, FloA and FloT (48). FloA and FloT physically interact (49), acting as scaffold proteins and thereby facilitating the interaction of other raft-associated proteins involved in signal transduction, protein secretion, and transport processes (50). Given the heterogeneous distribution of NisT throughout the cell membrane, we thus explored the association between the complex NisBTC and flotillins. The mKate2-labeled complex NisBTC was introduced in B. subtilis simultaneously and chromosomally integrated with FloA-sfGFP under the control of the native promoter P*_nat_*. Colocalization analyses indicated that NisBTC was associated with FloA, particularly for relatively bright foci, to a certain extent (Pearson’s correction coefficient, *r* = 0.78) (Fig. 8A). To examine the mobility of the complex NisBTC, we performed time-lapse microscopy to visualize the bacteria expressing mKate2-tagged NisBTC with a frame rate of 10 s. NisBTC foci were found to be immobile, and there was even no change in intensity (Fig. 8B). The flotillin protein FloA was shown to be highly dynamic in the cell membrane (Fig. 8C). However, when incorporating the nisin expression system, the movement of FloA foci within the membrane was significantly inhibited, and even the foci of FloA became quite stable (Fig. 8D). With regard to the coupling of the colocalization of NisBTC with FloA, it is tempting to speculate that the abundant assembly of the complex NisBTC brings a heavy burden to lipid rafts, and thus, the mobility of lipid rafts is severely decreased.

## DISCUSSION

Previously, the efforts to produce nisin in B. subtilis were made by incorporating the nisin gene cluster *nisABTCIPRKFEG* into the chromosome (51). Although the mRNA transcripts of all the genes from the cluster were observed by reverse transcription PCR (RT-PCR), the production of nisin was not detected. In this case, the original RBSs of nisin-related genes were probably not properly recognized by the translation machinery of B. subtilis. We also encountered the same problem when integrating the operon *nisABTC* into the B. subtilis chromosome. After replacement of the RBSs, fully modified precursor nisin was produced and secreted into the medium by WB800 and PG10, and thus, the production platform of precursor nisin in B. subtilis was established. When we performed the labeling approach with fluorescent proteins, with which all the proteins can be labeled and produced, their function or interaction with partner proteins may be hampered by the ∼26-kDa tags. Thus, both N- and C-terminal fusions were created, and a polyglycine linker was employed to join the fluorescent protein with the target protein to avoid steric interference. Finally, we screened out the fusion proteins that were functionally active and exhibited good signals in the B. subtilis cells, ensuring that all the data on the localization associated with the nisin biosynthetic machinery are reliable.

Our data indicate that NisA displays a homogeneous distribution throughout the cells in accordance with our expectation. Two-color fluorescence microscopy showed that NisB and NisC are colocalized with NisT in discrete foci distributed in the cell membrane, generating a functional nisin modification and transport machinery NisBTC, which is reinforced by the successful isolation of the NisBTC complex from the membrane fraction of B. subtilis cells by a pulldown assay with His-tagged NisT. To our knowledge, this is the first study to demonstrate the direct visualization of the wild-type lantibiotic biosynthesis complex *in vivo*, simultaneously verifying the presence of the putative machinery NisBTC. Nevertheless, as we described in a previous study, NisBTC is assumed to be assembled mainly at old cell poles in L. lactis (26), quite distinguishable from the punctate distribution in the membrane of B. subtilis. In fact, proteins, including membrane-associated machineries, often tend to localize in various patterns in different Gram-positive bacteria, particularly distinct in coccoid- and rod-shaped bacteria. For instance, the translocons of the Sec secretion system in B. subtilis have been shown to localize in spirals along the cytoplasmic membrane (29). However, in Streptococcus pyogenes, Sec secretion takes place in one specific area, the ExPortal (52, 53); in Streptococcus pneumoniae, Sec proteins primarily localize at the septum of predivisional cells and at one pole in postdivisional cells (54). In B. subtilis, TatA displays a dual localization pattern, being localized peripherally and showing bright foci which are predominantly located at the division sites and/or poles of the cells (31). The ABC transporter LmrB of L. lactis, dedicated to the transport of a small antimicrobial peptide, is distributed all around the cytoplasmic membrane (55). In short, the different localization pattern of the nisin biosynthesis machinery heterologously expressed in B. subtilis from that in L. lactis indicates that they employ different assembly sites within the cells. The foci of the complex NisBTC are easily visible and relatively bright in B. subtilis in comparison to the transient assembled complex in L. lactis. The reason for that is probably the lower transportation efficiency in the heterologous host. In B. subtilis cells, intracellular NisA accumulates continually, followed by the formation of more nisin modification machinery. We propose that abundant complexes of NisBTC are formed within the membrane when the modification machinery with modified substrate encounters the transporter.

When NisA and NisT were deficient, NisB and NisC were observed to be localized at the cell poles and septum in B. subtilis, consistent with the polar localization of NisBC in L. lactis, suggesting a potentially universal fact that the modification of the peptide mostly occurs at the poles of bacterial cells. As soon as the expression of NisA and NisT was induced, NisB and NisC were found to be recruited from the cell poles and septum to NisT that was distributed throughout the membrane, resulting in the complex NisABTC restricted to discrete foci along the cell periphery. The redistribution of proteins within cells has been reported in numerous studies. The *liaIH* operon of B. subtilis is the main target of the envelope stress-inducible two-component system LiaRS. Under noninducing conditions, LiaI locates in highly motile membrane-associated foci, while LiaH is dispersed throughout the cytoplasm. Under stress conditions, both proteins are strongly induced and colocalize in numerous distinct static spots at the cytoplasmic membrane (39). In E. coli, yellow fluorescent protein (YFP)-tagged TatA is located predominantly in bright foci within the cell membrane. Coexpression with TatE caused a striking redistribution of fluorescence, with the majority of the fluorescent proteins now present as a dispersed halo around the periphery of the cells with only occasional bright foci (56). Importantly, the remarkable change of NisBC localization over time exposes the initial process of NisBTC assembly. In our study performed in L. lactis, the NisBTC assembly was mediated by the recruitment of the “empty” NisT to NisBC largely confined to the old cell poles (26). The assembly process seems to be reversed in B. subtilis and L. lactis. However, we found, in the absence of NisA, NisB, and NisC, that NisT alone displayed a homogeneous distribution without any bright foci within the cell membrane, obviously different from the punctate localization when the machinery was intact. Coexpression with NisB and/or NisC also caused a rearrangement of the distribution with NisT present as independent foci around the periphery of the cells. Together, the difference in NisT distribution pattern revealed a recruitment behavior of NisT to NisBC that has been incorporated into the complex NisBTC.

**FIG 9:**
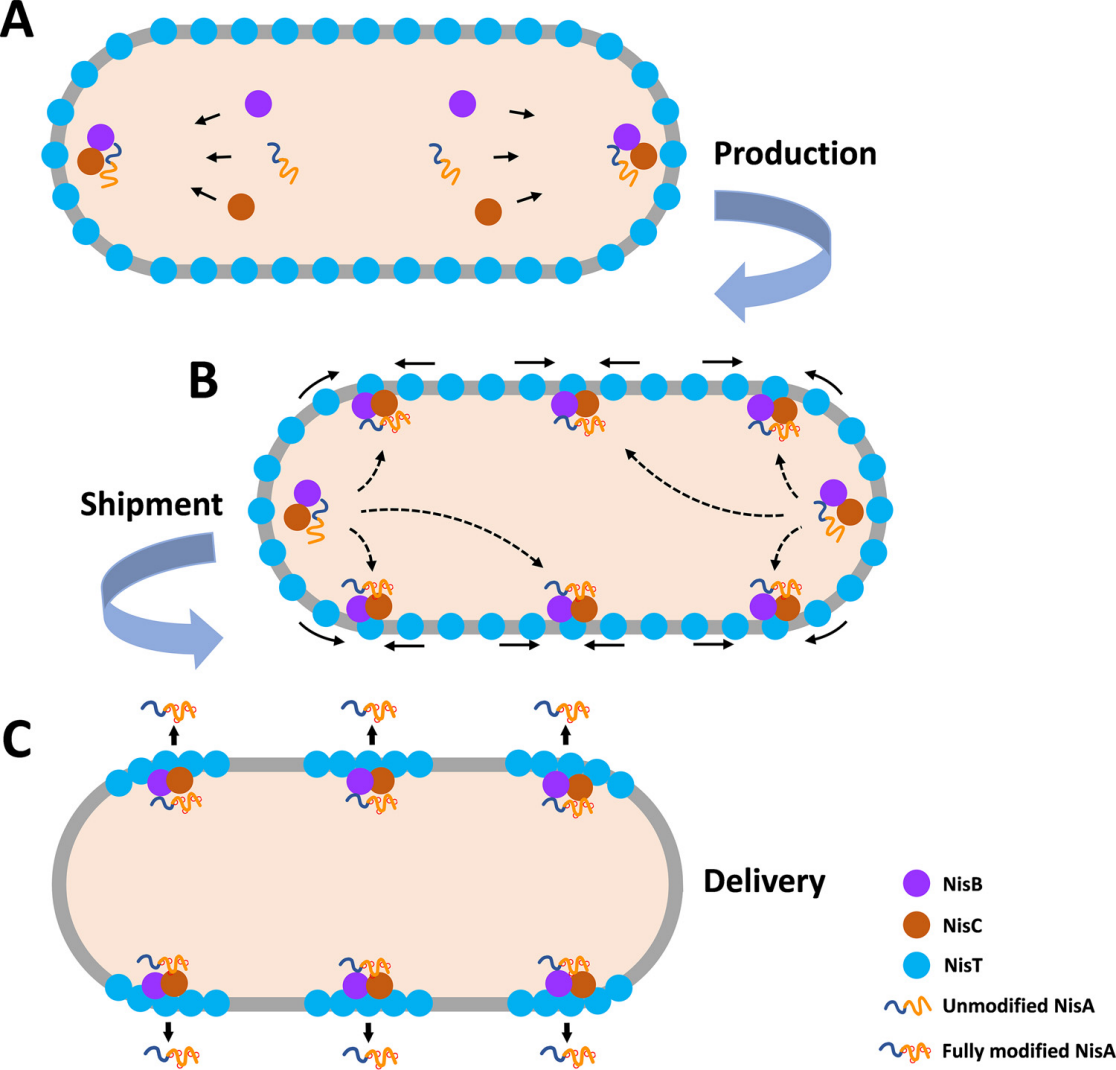
Proposed model for the three-phase production of precursor nisin in B. subtilis. (A) Modification machinery composed of NisA, NisB, and NisC is formed at the cell poles and septum. NisA is modified by NisB and NisC consecutively. This is the production phase. (B) NisBC in complex with NisA being modified is recruited to NisT that is homogeneously distributed across the membrane via the interaction with the NBD domain, initiating the first-step assembly of the complex NisBTC. Next, NisT near the assembled NisBTC within the membrane is driven to the complex by NisBC with an unbound leader peptide which becomes freely accessible, completing the second-step assembly of NisBTC. The microdomain where the functional nisin biosynthesis machinery is concentrated is therefore constructed. This could be regarded as the shipment phase. (C) Once the five (methyl-)lanthionine rings are formed, NisA is released from NisBC and binds to NisT. Finally, the fully modified precursor nisin is exported outside the cells. This is the actual delivery phase.

Hence, we propose a model for the three-phase production of fully modified precursor nisin in B. subtilis (Fig. 9). The leader peptide of the ribosomally synthesized and diffused NisA in the cytoplasm is recognized by NisB and NisC, later on forming the modification complex at the cell poles or septum to proceed with the dehydration and cyclization reactions in the portion of the core peptide (Fig. 9A). This could be regarded as the production factory. Meanwhile, unloaded NisT is homogeneously distributed in the cell membrane. Subsequently, NisBC, in complex with NisA, which has been modified or is being modified, is targeted to NisT via interaction with the NBD domain, initiating the first-step assembly of NisBTC and laying the foundations of export site formation for precursor nisin. As soon as the early complex NisBTC is installed, the targeting of “empty” NisT to the machinery is triggered: NisT is localized peripherally in the B. subtilis membrane, diffuses laterally in the membrane, and is captured by membrane-anchoring NisBC with an unbound leader peptide, which becomes freely accessible, from the membrane close to the assembled export sites. In this stage, the modification of NisA would be finished, and NisA (fully modified precursor nisin with leader peptide attached) is in a translocation-component state (Fig. 9B). This is the so-called second-step assembly to promote the aggregation of the NisBTC complex, which is similar to the model of the polar assembly process of NisBTC in L. lactis. The two-step assembly process can be regarded as the shipment phase. Afterward, due to completion of all (methyl-)lanthionine rings, fully modified NisA is released from the complex NisBC (17) and handed over to the dedicated transporter system NisT, which is associated with NisBC, to be exported outside the cells (Fig. 9C). This we could see as the actual delivery phase. The movement behavior that, regardless of whether NisBC is recruited to NisT or NisT is driven to NisBC, implies a crucial role for NisBC in the highly efficient secretion of precursor nisin and a mechanism by which the premature secretion of unfinished precursor nisin is prevented. Although NisT has been reported to be able to transport unmodified precursor nisin when NisB and NisC are deficient, the yield of secreted unmodified peptide is extremely low. The transport efficiency by NisT is markedly enhanced in the presence of NisB. When both NisB and NisC are coexpressed, NisT retained full transport ability (57). In our study, not only a specific interaction of NisT with NisC but also a direct interaction between NisT and NisB were identified. Even when NisA is absent, a stable complex of NisT with NisBC is present in the cell membrane, as our results show. Therefore, NisBC might play as a courier to deliver NisA to unloaded NisT beyond the function of peptide modification. The binding affinity of NisB to fully modified NisA is lower than that to dehydrated or unmodified NisA (23). The *in vitro* assembly assay of NisABC suggests that NisA is released from NisBC once the fifth lanthionine ring is created (17). Due to a higher affinity to NisBC, NisA being modified escapes binding to NisT, in consequence, avoiding the transport of uncompleted NisA. As the secretion of nisin has been suggested to follow a channeling mechanism (57), we hypothesize that the binding of NisBC to the NBD domain triggers a conformational transition and promotes the opening of the channel of NisT so that the released fully modified NisA could bind. The binding of ATP to the NBD domain of NisT changes the conformation of NisT, leading to the transport of fully modified NisA, and meanwhile breaks the interaction with NisBC, facilitating a new transport cycle.

In contrast with the single-export microdomain located mainly at the old cell poles in L. lactis, multiple secretion sites for lantibiotics were observed in B. subtilis, distributed along the cell periphery, in particular along the longitudinal axis. Perhaps there is more space at these locations than at the very crowded poles and septum where many other important processes take place. Especially in rod-shaped cells, the cell poles constitute important platforms for cellular regulation that underlie processes as essential as cell cycle progression, cellular differentiation, competence, virulence, chemotaxis, and growth of appendages (58). Owing to multiple export sites, the secretion efficiency of lantibiotics can be maintained at a high level. For instance, the Sec translocases display a pattern of punctate and spiral distribution in B. subtilis (29). Additionally, lipid rafts probably assist in the assembly of the complex NisBTC and are related to the heterogeneity of its distribution within the cell membrane, as suggested in this study. In B. subtilis, among the proteins copurified with the flotillin marker FloT, many are involved in transport processes (49). In Borrelia burgdorferi, lipid rafts are rich in proteins associated with binding/transport, especially ABC transporters (59). In Staphylococcus aureus, flotillin scaffold activity contributes to type VII secretion system assembly (47). The detailed interactions between lipid rafts and the lantibiotic biosynthetic machinery remain to be investigated. Future experiments isolating the complex NisABTC coupling with lipid rafts-related proteins will create direct insights into the assembly process of the machinery presumably located in the functional membrane microdomain.

In conclusion, we have established a platform for the production of precursor nisin in B. subtilis and have demonstrated the direct visualization of the nisin biosynthesis machinery. The complex NisBTC, with or without NisA, is distributed in discrete foci along the cell periphery, which is in line with the two-step assembly model that we proposed here. The interactions among the machinery-associated components were characterized systematically, emphasizing the importance of the association of NisBC with NisT for the efficient production of precursor nisin.

## MATERIALS AND METHODS

### Bacterial strains and growth conditions.

Bacterial strains of E. coli and B. subtilis and plasmids used in this study are listed in Table S3 in the supplemental material, respectively. The bacterial strain L. lactis NZ9700 was used as the source of nisin biosynthetic genes. Micrococcus flavus was employed as the indicator strain for the detection of modified nisin. E. coli DH5α served as a host for cloning and plasmid preparation. Both B. subtilis and E. coli were grown in Luria-Bertani (LB) medium at 37°C under aerobic conditions (with shaking at 220 rpm). The antibiotics were added when necessary, as follows: 100 μg/ml ampicillin for E. coli, 100 μg/ml spectinomycin, 0.5 μg/ml erythromycin, or 12.5 μg/ml lincomycin for B. subtilis. For induction in B. subtilis, 0.5% (wt/vol) xylose or 0.1 mM IPTG was added into the medium to initiate the expression of genes under the control of P*_xylA_* or P*_hy_spank_*. We added 1.5% (wt/vol) agar to the growth medium for solid medium. All chemicals were purchased from Sigma-Aldrich.

10.1128/mBio.01219-21.8 TABLE S3 Strains and plasmids used in this study. Download Table S3, DOCX file, 0.03 MB. Copyright © 2021 Chen et al. 2021 Chen et al. https://creativecommons.org/licenses/by/4.0/ This content is distributed under the terms of the Creative Commons Attribution 4.0 International license.

### Recombinant DNA techniques and oligonucleotides.

The techniques of standard molecular cloning were performed as described previously (60). The GenElute genomic DNA kit (Sigma-Aldrich, St. Louis, MO) was used to isolate genomic DNA of L. lactis. The NucleoSpin Plasmid EasyPure kit (Bioke, Leiden, the Netherlands) and the NucleoSpin gel and PCR clean-up kit (Bioke, Leiden, the Netherlands) were employed to extract plasmids and purify PCR products following the manufacturer’s instructions, respectively. PCRs were conducted with PrimeStar Max DNA polymerase (TaKaRa Bio Europe SAS, Saint-Germain-en-Laye, France) referring to the manufacturer’s protocol. The obtained PCR products were mixed and treated with the Gibson Assembly master mix (Bioke, Leiden, the Netherlands), yielding 20-nucleotide overhangs annealing to complementary overhangs. The mixtures were applied to transform E. coli DH5α directly to generate plasmids. Oligonucleotides used in this work were purchased from Biolegio BV (Nijmegen, the Netherlands) and are given in Table S4. The transformation of E. coli strains was performed following the standard procedures (60). B. subtilis 168 and WB800 were transformed based on natural competence (61). In mini*Bacillus* PG10, competence genes (*comK* and *comS*) controlled by the mannitol-inducible promoter (P*_mtlA_*) were induced by adding 0.5% (wt/vol) mannitol (62). All nucleotide sequencing was performed at Macrogen Europe (Amsterdam, the Netherlands). The detailed procedure of all the plasmid constructions is described in Text S1.

10.1128/mBio.01219-21.9 TABLE S4 Oligonucleotides used in this study. Download Table S4, DOCX file, 0.01 MB. Copyright © 2021 Chen et al. 2021 Chen et al. https://creativecommons.org/licenses/by/4.0/ This content is distributed under the terms of the Creative Commons Attribution 4.0 International license.

### Trichloroacetic acid precipitation.

B. subtilis was grown overnight in LB medium with appropriate antibiotics. The overnight culture was 2% diluted in fresh LB medium and was grown at 37°C. When optical density at 600 nm (OD_600_) was 0.8, 0.5% (wt/vol) xylose and 0.1 mM IPTG were added. Cell culture was collected at the stationary phase. We added 100% (wt/vol) trichloroacetic acid (TCA) to 45 ml culture supernatant with a final concentration of 10% (wt/vol) TCA. The mixture was kept on ice for 2 h and then centrifuged at 10,000 × *g* for 60 min at 4°C. The pellet was retained after discarding the supernatant. Subsequently, one-half original volume of iced acetone was added to the pellet. After 60 min centrifugation at 10,000 × *g* again, the pellet was retained and dried by vacuum freezing desiccation. Finally, the dry pellet was resuspended in 0.5 ml 50 mM Tris-HCl, pH 7.0.

### Antimicrobial activity assay.

Micrococcus flavus was used as indicator strain and grown overnight in liquid M17 medium supplemented with 0.5% (wt/vol) glucose (GM17) under aerobic conditions. One hundred microliters of diluted culture (OD_600_, 0.5) was added to 100 ml melted GM17 agar at 45°C and poured into plates. We dropped 10 μl sample with the addition of 1 μl purified protease NisP (Lab stock) on the plate after the agar was solid. The plates were left overnight at 30°C.

### Mass spectrometry analysis.

One microliter of each sample was spotted, dried, and washed with Milli-Q water on the target. Subsequently, 1 μl of 5 mg/ml α-cyano-4-hydroxycinnamic acid (Sigma-Aldrich) was spotted on the top of the samples. An ABI Voyager DE-Pro (Applied Biosystems) matrix-assisted laser desorption ionization–time of flight analyzer (MALDI-TOF) operating in linear mode using external calibration was used to obtain mass spectra.

### Cell fractionation.

The overnight culture of B. subtilis was 2% inoculated in fresh LB medium and grown at 37°C. When OD_600_ was 0.8, 0.5% (wt/vol) xylose and 0.1 mM IPTG were added. Cells were grown for 3 h and harvested by centrifugation. The cell pellet was washed with 50 mM Tris-HCl (pH 7.4), resuspended in cell lysis buffer (50 mM NaH_2_PO_4_, 300 mM NaCl, pH 8.0) with 10 mg/ml (wt/vol) lysozyme and protease inhibitor, and incubated for 60 min at 37°C. The cells were disrupted by French press machine. The obtained lysate suffered centrifugation to remove cell debris. The supernatant was ultracentrifuged (40,000 × *g* for 1 h, 4°C) and the new supernatant (cytoplasmic fraction) was collected again. The membrane pellet was resuspended in cell lysis buffer and ultracentrifuged again (40,000 × *g* for 30 min, 4°C). Finally, the collected membrane fraction was resuspended in the lysis buffer. Bicinchoninic acid (BCA) reagent was used to determine the protein concentrations of all collected fractions, and 30 μg total protein was loaded per lane when SDS-PAGE was performed.

### Membrane protein purification.

A standard procedure (nickel-nitrilotriacetic acid [Ni-NTA] purification) was followed and conducted in the cold room (4°C) to purify the membrane protein. Collected membrane fraction after ultracentrifugation was resuspended in binding buffer (50 mM NaH_2_PO_4_, 300 mM NaCl, 10 mM imidazole, pH 8.0). The total membrane protein concentration was measured by BCA assay (Thermo Fisher Scientific). Membranes were solubilized with 1% (wt/vol) *n*-dodecyl-β-d-maltoside (DDM) for 2 h at 4°C. Insoluble material was removed by ultracentrifugation at 40,000 × *g* for 30 min. Five milliliters binding buffer with 0.1% (wt/vol) DDM was run over the column containing Ni-NTA agarose (50%, 1.0 ml; Qiagen Benelux B.V.) to equilibrate it. Subsequently, 10 ml of the soluble membrane flowed through the column material twice to allow His-tagged protein to bind to the Ni-NTA agarose. Next, the column material was washed twice with 10 ml wash buffer (50 mM NaH_2_PO_4_, 300 mM NaCl, 20 mM imidazole, 0.1% [wt/vol] DDM, pH 8.0). Elutions were collected in 5 fractions (0.5 ml each) using elution buffer (50 mM NaH_2_PO_4_, 300 mM NaCl, 250 mM imidazole, 0.1% [wt/vol] DDM, pH 8.0). Finally, purified proteins were analyzed by SDS-PAGE and Western blotting.

### SDS-PAGE and Western blotting.

The samples for SDS-PAGE were incubated in loading buffer containing 5% (vol/vol) β-mercaptoethanol and boiled for 10 min. SDS-PAGE was performed according to a standard operation manual (60). Western blotting was performed using antileader peptide, anti-NisB, anti-NisC, or anti-GFP antibodies.

### Sample preparation for microscopy.

B. subtilis was grown overnight at 37°C in LB medium supplemented with appropriate antibiotic from freshly isolated colonies on plate. The overnight culture was diluted in LB medium to an OD_600_ of 0.05 and grown at 37°C. When OD_600_ reached 0.8, 0.5% (wt/vol) xylose and/or 0.1 mM IPTG were added. When the timed expression was performed, first, 0.5% (wt/vol) xylose was added to the culture at OD_600_ of 0.8, while 0.1 mM IPTG was added after 30 min xylose induction. Cells containing sfGFP or mKate2-labeled proteins for microscopic observation were taken at exponential phase. Cell membrane was visualized with Nile red. After washing the cells using phosphate-buffered saline (PBS; pH 7.4), the cells were immobilized on agarose (1.0% [wt/vol])-coated microscope slides to be examined.

### Fluorescence microscopy.

All micrographs were captured using a DeltaVision Elite inverted epifluorescence microscope (Applied Precision, GE Healthcare, Issaquah, WA, USA) equipped with a stage holder, a climate chamber, a seven-color combined set InsightSSI solid-state illumination module, and a scientific complementary metal oxide semiconductor (sCMOS) camera (PCO AG, Kelheim, Germany). A 100× phase-contrast objective (numerical aperture [NA], 1.4; oil immersion, DV) was used for image capturing, in combination with SoftWoRx 3.6.0 software (Applied Precision) to control the microscope setup and to perform single-time point or time-lapse imaging of cells. The following standard fluorescence filter sets were used for visualization: for sfGFP, excitation at 475/28 nm and emission at 525/48 nm, and for mKate2, excitation at 573.5/33 nm and emission at 607.5/19 nm. For time point microscopy, a standard microscope slide was prepared with a layer of solidified agarose (1% [wt/vol], in appropriate medium), and 1 μl of bacterial cells was loaded onto the agarose. The sample was covered with a standard microscope coverslip for microscopic observations. For time-lapse microscopy, microscope slides were incubated in the temperature-controlled (cube and box incubation system; Life Imaging Services) automated microscope (DeltaVision Elite) at 37°C. Images were captured at 10-s intervals and the XYZ position stored in the microscope control software SoftWoRx.

### Data analysis of microscopy images.

Images were deconvolved with the SoftWorks imaging software. Color assignment and overlay images were created using ImageJ software (https://imagej.net/Fiji) and saved as green/red tagged-image file format (TIFF) files. The fluorescence profile within cells was analyzed by ImageJ plug-in Plot Profile. The ImageJ-based ScatterJ was employed to determine Pearson’s correlation coefficient (PCC) (63). Prior to ScatterJ analysis, the images were processed with a discoidal averaging filter to increase the signal-to-noise ratio of the detected foci and to remove all background signals. The PCC is a well-established measure of correlation and has a range of +1 (perfect correlation) to −1 (perfect but negative correlation), with 0 denoting the absence of a relationship. All different images were acquired with the same exposure time. Image processing consists of equivalent adjustments of brightness and contrast on complete images. Gamma and lookup table (LUT) values were not modified and were left as linear on each channel. In this study, all the experiments were repeated at least 3 times.
