# Supplementary figures and images for: Visualization and Analysis of the Dynamic Assembly of a Heterologous Lantibiotic Biosynthesis Complex in Bacillus subtilis
Source: mBio. 2021 Jul 20;12(4):e01219-21. doi: 10.1128/mBio.01219-21 (PMC8406302; doi:10.1128/mBio.01219-21)

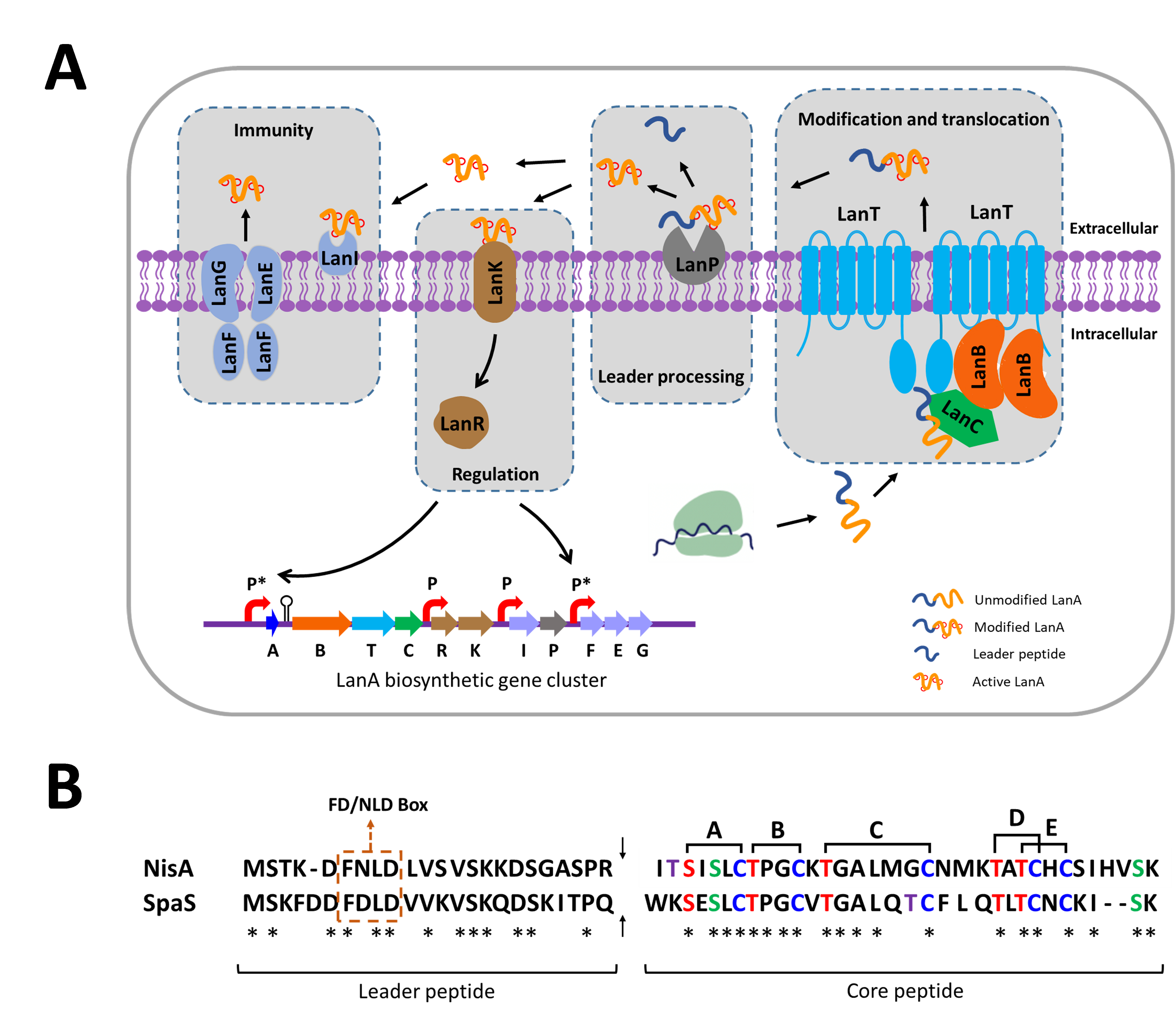

Supplement: FIG S1 [file mbio.01219-21-sf001.tif]

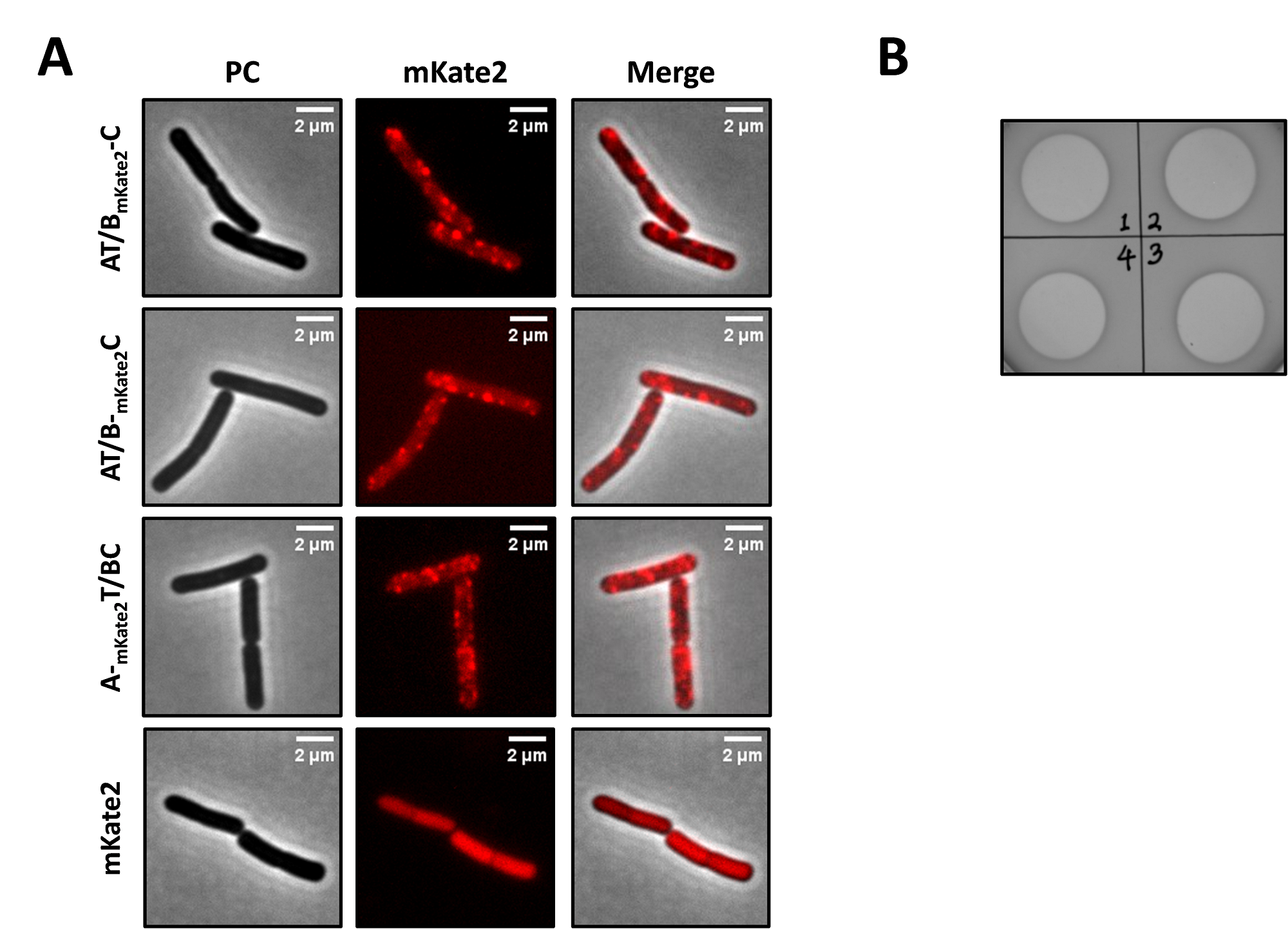

Supplement: FIG S2 [file mbio.01219-21-sf002.tif]

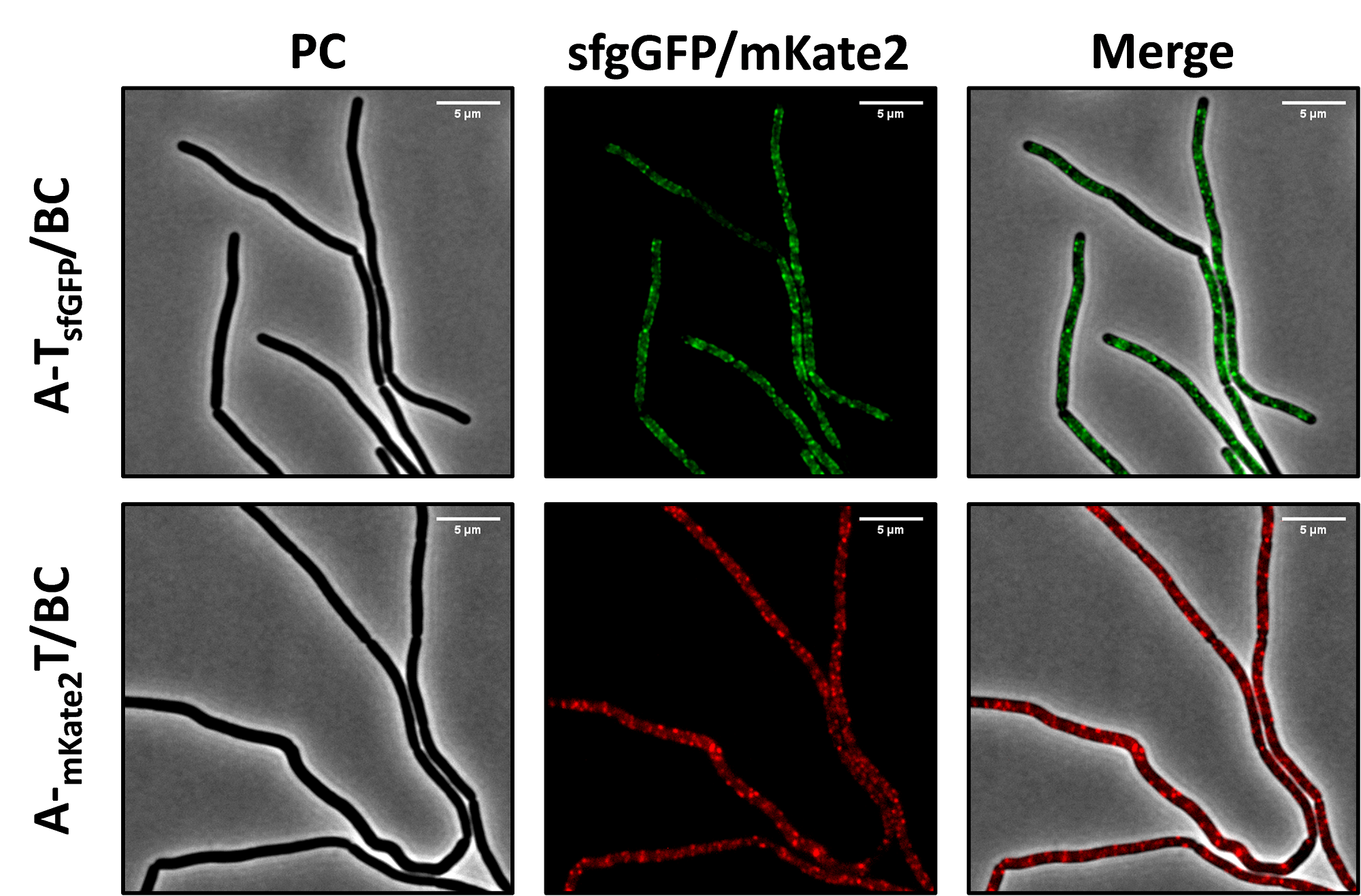

Supplement: FIG S3 [file mbio.01219-21-sf003.tif]

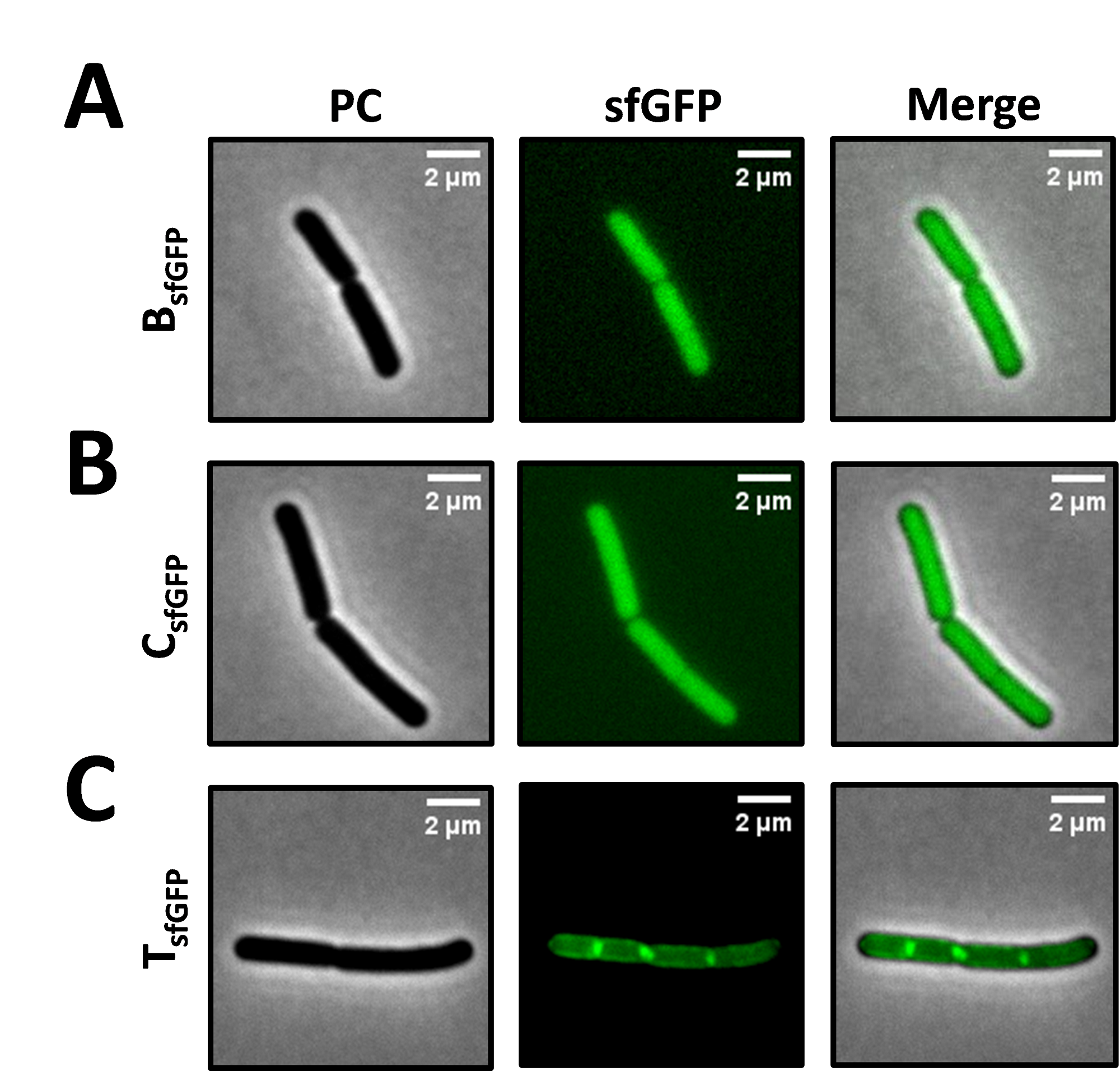

Supplement: FIG S4 [file mbio.01219-21-sf004.tif]

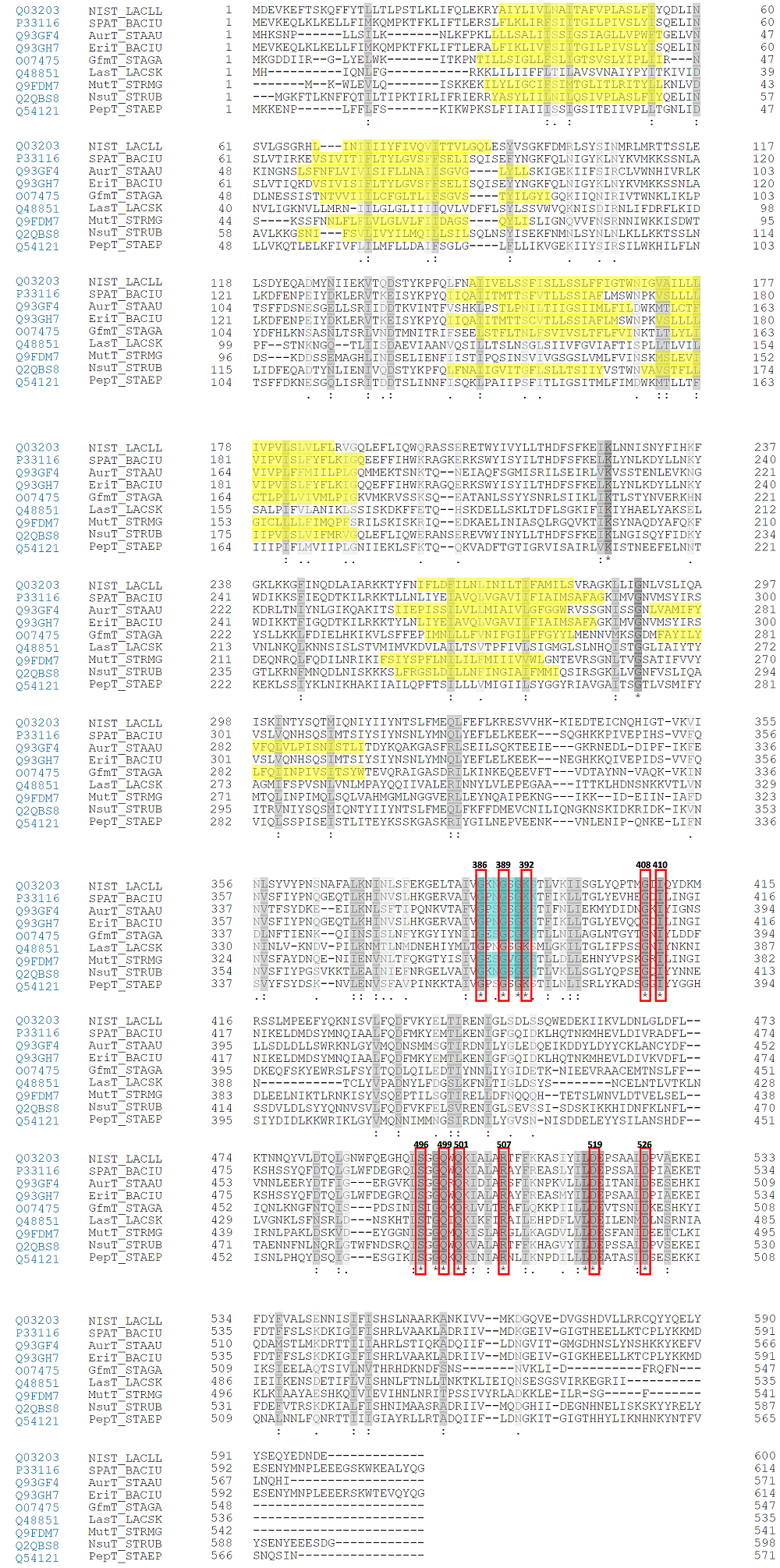

Supplement: FIG S5 [file mbio.01219-21-sf005.tif]
