# Supplementary material for: Visualization and Analysis of the Dynamic Assembly of a Heterologous Lantibiotic Biosynthesis Complex in Bacillus subtilis
Source: mBio. 2021 Jul 20;12(4):e01219-21. doi: 10.1128/mBio.01219-21 (PMC8406302; doi:10.1128/mBio.01219-21)
Supplement: TABLE S1 [file mbio.01219-21-st001.docx]

**Table S1** Similarity and identity between the LanA, LanB, LanC, and LanT proteins of the nisin and subtilin systems.

| Protein | Bacteria | Amino acid | Size (kDa) | Identity |
| --- | --- | --- | --- | --- |
| NisA | *Lactococcus lactis* | 57 | 6.0 | 50% |
| SpaS | *Bacillus subtilis* | 56 | 6.2 |  |
| NisB | *Lactococcus lactis* | 993 | 117.5 | 27.2% |
| SpaB | *Bacillus subtilis* | 1030 | 120.5 |  |
| NisC | *Lactococcus lactis* | 418 | 47.9 | 26.5% |
| SpaC | *Bacillus subtilis* | 441 | 49.3 |  |
| NisT | *Lactococcus lactis* | 600 | 69.2 | 42.4% |
| SpaT | *Bacillus subtilis* | 614 | 71.2 |  |
| NisP^a^ | *Lactococcus lactis* | 682 | 74.8 | - |
| AprE/WprA/Vpr/Bpr^b^ | *Bacillus subtilis* | - | - |  |

^a^ The serine protease used for specifically removing the leader peptide of precursor nisin in *L. lactis*.

^b^ The subtilin system doesn’t contain a specific protease for leader peptide cleavage. Four extracellular proteases of *B. subtilis* (AprE, WprA, Vpr, and Bpr) were found to involve in subtilin leader peptide processing.

-, not assessed.
