# Supplementary material for: Visualization and Analysis of the Dynamic Assembly of a Heterologous Lantibiotic Biosynthesis Complex in Bacillus subtilis
Source: mBio. 2021 Jul 20;12(4):e01219-21. doi: 10.1128/mBio.01219-21 (PMC8406302; doi:10.1128/mBio.01219-21)
Supplement: TEXT S1 [file mbio.01219-21-s0001.docx]

**Text S1 Materials and Methods**

Nucleotide sequences of the primers described below are presented in **Table S4**. Pertinent regions of all plasmids were sequenced to confirm their proper nucleotide sequences. All strains and plasmids that were used or created during this study are given in **Table S3**.

**Strain Construction**

The integration plasmids deriving from pDR111 and pDG1664 were constructed in *Escherichia coli* DH5α and subsequently transferred to *Bacillus subtilis* 168, WB800 or PG10. Prior to transformation, the integration plasmids were digested by single restriction enzyme, generating linear DNA. Double-crossover reaction occurred and a series of recombinant *B. subtilis* strains were screened out with appropriate resistance. All the resulting strains contain an insertion of corresponding genes into the *thrC* or *amyE* locus depending on the integration plasmid. Plasmids were transferred to component cells of *B. subtilis* 168 and WB800 based on natural competence ([1](#_ENREF_1)). For PG10, competence genes (*comK* and *comS*) controlled by the mannitol-inducible promoter (P*_mtlA_*) were induced by adding 0.5% (w/v) mannitol ([2](#_ENREF_2)).

**Plasmid Construction**

pDG1664-*sfgfp* and pDG1664-*mKate2* were created by cloning the DNA fragments *sfgfp* and *mKate2* into the vector pDG1664, respectively, using Gibson Assembly strategy ([3](#_ENREF_3)). The gene *sfgfp* was amplified from pUC57-*sfgfp* using primers PC01 and PC02. The gene *mKate2* was amplified using pSEUDO-P*_usp45_*-*mKate2* as template with primer pair PC03/PC04. The linear vector was obtained from pDG1664 by PCR using the primers PC05 and PC06. In the constructed plasmids, *sfgfp* or *mKate2* was under the control of xylose inducible promoter P*_xylA_*.

To achieve heterologous expression of precursor nisin in *B. subtilis*, plasmids pDR111-*nisAT* and pDG1664-*nisBC* were constructed. pDR111-*nisAT* was made by two steps: firstly, the gene *nisT* was amplified from *L. lactis* NZ9700 genomic DNA using primers PC07 and PC08. The linear vector backbone was amplified from the template pDR111 using the primer pairs PC09/PC10. The amplicon was inserted into pDR111 downstream of the promoter P*_hy_spanK_* using Gibson Assembly strategy, resulting into an intermediate plasmid pDR111-*nisT*. Next, using the primers PC11 and PC12, the gene *nisA* was cloned from NZ9700 genomic DNA. The linear vector was obtained based on pDR111-*nisT* by PCR using the primers PC10 and PC13. The DNA fragment *nisA* was then incorporated into pDR111-*nisT* between the promoter P*_hy_spank_* and the gene *nisT*, creating pDR111-*nisAT*. In pDR111-*nisAT*, the RBS sequence 5’-AAATCAAAGGGGGAAATCAT-3’ is located in front of *nisA*, and the RBS sequence 5’-CACCTAAAAAGGAGCGATTTACAT-3’ is located upstream of *nisT*. Similarly, pDG1664-*nisBC* was also constructed by two steps. The DNA insert *nisB* was amplified from NZ9700 chromosomal DNA using the primers PC14/PC15. The linear vector backbone was cloned from the plasmid pDG1664 using the primers PC05 and PC06. *nisB* was inserted into pDG1664 behind the promoter P*_xylA_* to get the intermediate plasmid pDG1664-*nisB*. Then, using the primers PC16 and PC17, *nisC* was obtained employing NZ9700 genomic DNA as template. The linear vector deriving from pDG1664-*nisB* was amplified using primer pairs PC05/PC18. Finally, the plasmid pDG1664-*nisBC* was generated by inserting *nisC* into pDG1664-*nisB* downstream of the gene *nisB* via Gibson Assembly strategy. In the plasmid pDG1664-*nisBC*, the RBS sequence 5’-AAATCAAAGGGGGAAATCAT-3’ is located in front of *nisB*, and the RBS sequence 5’-CACCTAAAAAGGAGCGATTTACAT-3’ is located upstream of *nisC*.

To fluorescently label precursor nisin NisA, pDR111-*nisA_sfgfp_-nisT* was created: the *sfgfp* gene was amplified from pUC57-*sfgfp* using primers PC19/PJ20. The linear vector was obtained by PCR from the constructed pDR111-*nisAT* using primers PJ21/PJ22. The amplicon was cloned behind the *nisA* gene with the deletion of stop codon into pDR111-*nisAT* to give the plasmid pDR111-*nisA_sfgfp_-nisT*. A flexible linker (5’-GGTAGCGGTGGAGGTGGCAGC-3’) was located between *nisA* and *sfgfp* to join them to reduce the interference to each other. Using the same strategy of the construction of pDR111-*nisA_sfgfp_-nisT*, pDR111-*nisA_mKate2_-nisT* (PC23/PC24 and PC21/PC22), pDR111-*nisA-_sfgfp_nisT* (PC25/PC26 and PC27/PC28), pDR111-*nisA-nisT_sfgfp_* (PC29/PC30 and PC09/PC31), pDR111-*nisA-_mKate2_nisT* (PC32/PC33 and PC27/PC28) and pDR111-*nisA-nisT_mKate2_* (PC34/PC35 and PC09/PC31) were created based on pDR111-*nisAT*. pDR111-*nisT_sfgfp_* was derived from pDR111-*nisA-nisT_sfgfp_* and obtained by deleting *nisA* using the primers PJ36 and PJ37. The gene *nisA* was removed from pDR111-*nisA-_mKate2_nisT* using the primers PJ38 and PJ39, leading to the plasmid pDR111-*_mKate2_nisT*. pDR111-*nisT^TMD^_sfgfp_* was made by deleting the NBD domain of NisT from pDR111-*nisT_sfgfp_* using the primer pair PC40/PC41. pDR111-*nisT^NBD^* and pDR111-*nisT^NBD^_sfgfp_* was generated by removing the TMD domain of NisT from pDR111-*nisT* and pDR111-*nisT_sfgfp_* using primer pairs PC42/PC43, respectively.

To conduct pull-down assay, pDR111-*nisA-nisT_His_* was made employing pDR111-*nisAT* as template and using the primers PC44 and PC45. 6xHis tag was fused to the C-terminus of NisT.

With the Gibson Assembly method, the plasmids pDG1664-*_sfgfp_nisB-nisC* (PC46/PC47 and PC48/PC49), pDG1664-*nisB_sfgfp_-nisC* (PC50/PC51 and PC52/PC53), pDG1664-*_mKate2_nisB-nisC* (PC54/PC55 and PC56/PC57), pDG1664-*nisB_mKate2_-nisC* (PC58/PC59 and PC60/PC61), pDG1664-*nisB-_sfgfp_nisC* (PC62/PC63 and PC64/PC65), pDG1664-*nisB-nisC_sfgfp_* (PC66/PC67 and PC68/PC69), pDG1664-*nisB-_mKate2_nisC* (PC70/PC71 and PC64/PC65) and pDG1664-*nisB-nisC_mKate2_* (PC72/PC73 and PC68/PC69) were made using pDG1664-*nisBC* as vector backbone. pDG1664-*nisB_sfgfp_* was constructed by removing *nisC* from pDG1664-*nisB_sfgfp_-nisC* using primers PC74 and PC75. In the same way, *nisB* was deleted from pDG1664-*nisB-nisC_sfgfp_* using the primers PC76 and PC77, leading to pDG1664-*nisC_sfgfp_*.

To use different fluorescent proteins to label the components of the nisin biosynthesis complex simultaneously, pDR111-*nisA_sfgfp_-_mKate2_nisT* and pDG1664-*nisB_sfgfp_- _mKate2_nisC* were created employing Gibson Assembly strategy. *sfgfp* was amplified using the primers PC78 and PC79. The linear vector was obtained by PCR from pDR111-*nisA-_mKate2_nisT* using the primers PC80 and PC81. *sfgfp* was inserted into pDR111-*nisA-_mKate2_nisT* immediately downstream of *nisA*, resulting in pDR111-*nisA_sfgfp_-_mKate2_nisT*. In the same way, pDG1664-*nisB_sfgfp_- _mKate2_nisC* was constructed using the primer pairs PC82/PC83 and PC84/PC85. Other strains containing dual fluorescently labeled proteins were constructed by combined integration of corresponding plasmids.

**References**

1. Harwood CR, Cutting SM. 1990. Molecular biological methods for *Bacillus*. Wiley, Chichester, New York.

2. Rahmer R, Morabbi Heravi K, Altenbuchner J. 2015. Construction of a super-competent *Bacillus subtilis* 168 using the P*_mtlA_*-c*omKS* inducible cassette. Front Microbiol 6:1431.

3. Gibson DG, Young L, Chuang RY, Venter JC, Hutchison CA, Smith HO. 2009. Enzymatic assembly of DNA molecules up to several hundred kilobases. Nature Methods 6:343-345.
