# Supplementary material for: Visualization and Analysis of the Dynamic Assembly of a Heterologous Lantibiotic Biosynthesis Complex in Bacillus subtilis
Source: mBio. 2021 Jul 20;12(4):e01219-21. doi: 10.1128/mBio.01219-21 (PMC8406302; doi:10.1128/mBio.01219-21)
Supplement: TABLE S2 [file mbio.01219-21-st002.docx]

**Table S2** Subcellular localization of NisB and NisC when conserved residues of NisT are mutated.

| **NisT** | **Subcellular localization** | |
| --- | --- | --- |
|  | **NisB** | **NisC** |
| No NisT | Cell pole/septum | Cell pole/septum |
| NisT | Membrane | Membrane |
| NisT^TMD^ | Cell pole/septum | Cell pole/septum |
| NisT^NBD^ | Cell pole/septum | Cell pole/septum |
| G386A | Membrane | Membrane |
| G389A | Membrane | Membrane |
| K392A | Membrane | Membrane |
| G408A | Membrane | Membrane |
| I410A | Membrane | Membrane |
| S496A | Membrane | Membrane |
| Q499A | Membrane | Membrane |
| Q501A | Membrane | Membrane |
| R507A | Membrane | Membrane |
| D519A | Membrane | Membrane |
| D526A | Membrane | Membrane |
