# Supplementary material for: Visualization and Analysis of the Dynamic Assembly of a Heterologous Lantibiotic Biosynthesis Complex in Bacillus subtilis
Source: mBio. 2021 Jul 20;12(4):e01219-21. doi: 10.1128/mBio.01219-21 (PMC8406302; doi:10.1128/mBio.01219-21)
Supplement: TABLE S3 [file mbio.01219-21-st003.docx]

**Table S3 Strains and plasmids used in this study**

| **Strains** | **Abbreviation** | | **Genotype** | **Source** |
| --- | --- | --- | --- | --- |
| *L. lactis* NZ9700 | NZ9700 | | *nisABTCIPRKFEG*, nisin producer | ([1](#_ENREF_1)) |
| *E. coli* DH5α | DH5α | | F^-^∆*lac*U169(Ø80d *lac*Z∆M15) *sup*E44 *hsd*R17 *rec*A1 *gyr*A96 *end*A1 *thi*-1 *rel*A1 | ([2](#_ENREF_2)) |
| *Micrococcus flavus* | - | | Indicator strain for nisin | ([3](#_ENREF_3)) |
| *B. subtilis* 168 | 168 | | *Trp^-^* | Lab stock |
| *B. subtilis* WB800 | WB800 | | Eight-fold protease-deficient strain; *ΔnprE ΔnprB ΔaprE Δepr Δmpr Δbpr Δvpr ΔwprA* | ([4](#_ENREF_4)) |
| *B. subtilis* PG10 | PG10 | | 168 derivative; large-scale genome-minimized strain | ([5](#_ENREF_5)) |
| BSJ01 | - | | 168 *thrC*::P*_xylA_*-*nisBC* | This study |
| BSJ02 | - | | 168 *thrC*::P*_xylA_*-*nisBC*, *amyE*:: P*_hy_spank_*-*nisT*; spec^r^, ery^r^ | This study |
| BSJ03 | - | | 168 *thrC*::P*_xylA_*-*nisBC*, *amyE*:: P*_hy_spank_*-*nisAT*; spec^r^, ery^r^ | This study |
| BSJ04 | BC | | WB800 *thrC*::P*_xylA_*-*nisBC* | This study |
| BSJ05 | T/BC | | WB800 *thrC*::P*_xylA_*-*nisBC*, *amyE*:: P*_hy_spank_*-*nisT*; spec^r^, ery^r^ | This study |
| BSJ06 | AT/BC | | WB800 *thrC*::P*_xylA_*-*nisBC*, *amyE*:: P*_hy_spank_*-*nisAT*; spec^r^, ery^r^ | This study |
| BSJ07 | - | | PG10 *thrC*::P*_xylA_*-*nisBC* | This study |
| BSJ08 | - | | PG10 *thrC*::P*_xylA_*-*nisBC*, *amyE*:: P*_hy_spank_*-*nisT*; spec^r^, ery^r^ | This study |
| BSJ09 | - | | PG10 *thrC*::P*_xylA_*-*nisBC*, *amyE*:: P*_hy_spank_*-*nisAT*; spec^r^, ery^r^ | This study |
| BSJ10 | A_sfGFP_-T/BC | | WB800 *thrC*::P*_xylA_*-*nisBC*, *amyE*:: P*_hy_spank_*-*nisA_sfgfp_-nisT*; spec^r^, ery^r^ | This study |
| BSJ11 | A_mKate2_-T/BC | | WB800 *thrC*::P*_xylA_*-*nisBC*, *amyE*:: P*_hy_spank_*-*nisA_mKate2_-nisT*; spec^r^, ery^r^ | This study |
| BSJ12 | AT/_sfGFP_B-C | | WB800 *thrC*::P*_xylA_*-*_sfgfp_nisB-nisC*, *amyE*:: P*_hy_spank_*-*nisAT*; spec^r^, ery^r^ | This study |
| BSJ13 | AT/B_sfGFP_-C | | WB800 *thrC*::P*_xylA_*-*nisB_sfgfp_-nisC*, *amyE*:: P*_hy_spank_*-*nisAT*; spec^r^, ery^r^ | This study |
| BSJ14 | AT/_mKate2_B-C | | WB800 *thrC*::P*_xylA_*-*_mKate2_nisB-nisC*, *amyE*:: P*_hy_spank_*-*nisAT*; spec^r^, ery^r^ | This study |
| BSJ15 | AT/B_mKate2_-C | | WB800 *thrC*::P*_xylA_*-*nisB_mKate2_-nisC*, *amyE*:: P*_hy_spank_*-*nisAT*; spec^r^, ery^r^ | This study |
| BSJ16 | AT/B-_sfGFP_C | | WB800 *thrC*::P*_xylA_*-*nisB-_sfgfp_nisC*, *amyE*:: P*_hy_spank_*-*nisAT*; spec^r^, ery^r^ | This study |
| BSJ17 | AT/B-C_sfGFP_ | | WB800 *thrC*::P*_xylA_*-*nisB-nisC_sfgfp_*, *amyE*:: P*_hy_spank_*-*nisAT*; spec^r^, ery^r^ | This study |
| BSJ18 | AT/B-_mKate2_C | | WB800 *thrC*::P*_xylA_*-*nisB-_mKate2_nisC*, *amyE*:: P*_hy_spank_*-*nisAT*; spec^r^, ery^r^ | This study |
| BSJ19 | AT/B-C_mKate2_ | | WB800 *thrC*::P*_xylA_*-*nisB-nisC_mKate2_*, *amyE*:: P*_hy_spank_*-*nisAT*; spec^r^, ery^r^ | This study |
| BSJ20 | A-_sfGFP_T/BC | | WB800 *thrC*::P*_xylA_*-*nisBC*, *amyE*:: P*_hy_spank_*-*nisA-_sfgfp_nisT*; spec^r^, ery^r^ | This study |
| BSJ21 | A-T_sfGFP_/BC | | WB800 *thrC*::P*_xylA_*-*nisBC*, *amyE*:: P*_hy_spank_*-*nisA-nisT_sfgfp_*; spec^r^, ery^r^ | This study |
| BSJ22 | A-_mKate2_T/BC | | WB800 *thrC*::P*_xylA_*-*nisBC*, *amyE*:: P*_hy_spank_*-*nisA-_mKate2_nisT*; spec^r^, ery^r^ | This study |
| BSJ23 | A-T_mKate2_/BC | | WB800 *thrC*::P*_xylA_*-*nisBC*, *amyE*:: P*_hy_spank_*-*nisA-nisT_mKate2_*; spec^r^, ery^r^ | This study |
| BSJ24 | sfGFP | | WB800 *thrC*::P*_xylA_*-*sfgfp*; spec^r^ | This study |
| BSJ25 | mKate2 | | WB800 *thrC*::P*_xylA_*-*mKate2*; spec^r^ |  |
| BSJ26 | A_sfGFP_-_mKate2_T/BC | | WB800 *thrC*::P*_xylA_*-*nisBC*, *amyE*:: P*_hy_spank_*-*nisA_sfgfp_-_mKate2_nisT*; spec^r^, ery^r^ | This study |
| BSJ27 | AT/B_sfGFP_-_mKate2_C | | WB800 *thrC*::P*_xylA_*-*nisB_sfgfp_-_mKate2_nisC*, *amyE*:: P*_hy_spank_*-*nisAT*; spec^r^, ery^r^ | This study |
| BSJ28 | A-_mKate2_T/B_sfGFP_-C | | WB800 *thrC*::P*_xylA_*-*nisB_sfgfp_-nisC*, *amyE*:: P*_hy_spank_*-*nisA-_mKate2_nisT*; spec^r^, ery^r^ | This study |
| BSJ29 | A-_mKate2_T/B-C_sfGFP_ | | WB800 *thrC*::P*_xylA_*-*nisB-nisC_sfgfp_*, *amyE*:: P*_hy_spank_*-*nisA-_mKate2_nisT*; spec^r^, ery^r^ | This study |
| BSJ30 | A-T_His_/BC | | WB800 *thrC*::P*_xylA_*-*nisBC*, *amyE*:: P*_hy_spank_*-*nisA-nisT_His_*; spec^r^, ery^r^ | This study |
| BSJ31 | _mKate2_T/B_sfGFP_-C | | WB800 *thrC*::P*_xylA_*-*nisB_sfgfp_-nisC*, *amyE*:: P*_hy_spank_*-*_mKate2_nisT*; spec^r^, ery^r^ | This study |
| BSJ32 | _mKate2_T/B-C_sfGFP_ | | WB800 *thrC*::P*_xylA_*-*nisB-nisC_sfgfp_*, *amyE*:: P*_hy_spank_*-*_mKate2_nisT*; spec^r^, ery^r^ | This study |
| BSJ33 | T/B_sfGFP_-_mKate2_C | | WB800 *thrC*::P*_xylA_*-*nisB_sfgfp_-_mKate2_nisC*, *amyE*:: P*_hy_spank_*-*nisT*; spec^r^, ery^r^ | This study |
| BSJ34 | B_sfGFP_-_mKate2_C | | WB800 *thrC*::P*_xylA_*-*nisB_sfgfp_-_mKate2_nisC*; spec^r^ | This study |
| BSJ35 | _mKate2_T/B_sfGFP_ | | WB800 *thrC*::P*_xylA_*-*nisB_sfgfp_*, *amyE*:: P*_hy_spank_*-*_mKate2_nisT*; spec^r^, ery^r^ | This study |
| BSJ36 | _mKate2_T/C_sfGFP_ | | WB800 *thrC*::P*_xylA_*-*nisC_sfgfp_*, *amyE*:: P*_hy_spank_*-*_mKate2_nisT*; spec^r^, ery^r^ | This study |
| BSJ37 | B_sfGFP_ | | WB800 *thrC*::P*_xylA_*-*nisB_sfgfp_*; spec^r^ | This study |
| BSJ38 | C_sfGFP_ | | WB800 *thrC*::P*_xylA_*-*nisC_sfgfp_*; spec^r^ | This study |
| BSJ39 | T_sfGFP_ | | WB800 *amyE*:: P*_hy_spank_*-*nisT_sfgfp_*; ery^r^ | This study |
| BSJ40 | T^NBD^_sfGFP_ | | WB800 *amyE*:: P*_hy_spank_*-*nisT^NBD^_sfgfp_*; ery^r^ | This study |
| BSJ41 | T^NBD^_sfGFP_/B-_mKate2_C | | WB800 *thrC*::P*_xylA_*-*nisB-_mKate2_nisC*, *amyE*:: P*_hy_spank_*-*nisT^NBD^_sfgfp_*; spec^r^, ery^r^ | This study |
| BSJ42 | T^TMD^_sfGFP_ | | WB800 *amyE*:: P*_hy_spank_*-*nisT^TMD^_sfgfp_*; ery^r^ | This study |
| BSJ43 | T^TMD^_sfGFP_/BC | | WB800 *thrC*::P*_xylA_*-*nisBC*, *amyE*:: P*_hy_spank_*-*nisT^TMD^_sfgfp_*; spec^r^, ery^r^ | This study |
| BSJ44 | T^NBD^/B_sfGFP_-_mKate2_C | | WB800 *thrC*::P*_xylA_*-*nisB_sfgfp_-_mKate2_nisC*, *amyE*:: P*_hy_spank_*-*nisT^NBD^*; spec^r^, ery^r^ | This study |
| BSJ45 | FloA_sfGFP_ | | WB800 *floA*::*floA_sfgfp_* | Lab stock |
| BSJ46 | FloA_sfGFP_/ A-_mKate2_T/BC | | WB800 *floA*::*floA_sfgfp_*, *thrC*::P*_xylA_*-*nisBC*, *amyE*:: P*_hy_spank_*-*nisA-_mKate2_nisT*; spec^r^, ery^r^ | This study |
| BSJ47 | FloA_sfGFP_/ AT/BC | | WB800 *floA*::*floA_sfgfp_*, *thrC*::P*_xylA_*-*nisBC*, *amyE*:: P*_hy_spank_*-*nisAT*; spec^r^, ery^r^ | This study |
| BSJ48 | - | | PG10 *thrC*::P*_xylA_*-*nisBC*, *amyE*:: P*_hy_spank_*-*nisA-nisT_sfgfp_*; spec^r^, ery^r^ | This study |
| BSJ49 | - | | PG10 *thrC*::P*_xylA_*-*nisBC*, *amyE*:: P*_hy_spank_*-*nisA-_mKate2_nisT*; spec^r^, ery^r^ | This study |
| **Plasmids** | | **Characteristics** | | **Source** |
| pDR111 | | Integration vector for genomic integration in the *amyE* locus of *B. subtilis*; IPTG inducible promoter P_hy__*_spank_*; *spec^r^*, *amp^r^* | | Lab stock |
| pDG1664 | | Integration vector for genomic integration in the *thrC* locus of *B. subtilis; xylR,* xylose inducible promoter P*_xylA_; ery^r^, spec^r^, amp^r^* | | Lab stock |
| pUC57-*sfgfp* | | *sfgfp,* encoding sfGFP*,* *amp^r^* | | Lab stock |
| pSEUDO-P*_usp45_*-*mKate2* | | *mKate2,* encoding mKate2, *ery^r^* | | Lab stock |
| pDG1664-*sfgfp* | | pDG1664 derivative; Xylose inducible promoter P*_xylA_, sfgfp, ery^r^, spec^r^, amp^r^* | | This study |
| pDG1664-*mKate2* | | pDG1664 derivative; Xylose inducible promoter P*_xylA_, mKate2, ery^r^, spec^r^, amp^r^* | | This study |
| pDR111-*nisT* | | pDR111 derivative; IPTG inducible promoter P*_hy_*___*_spank_*, *nisT*, *spec^r^*, *amp^r^* | | This study |
| pDR111-*nisAT* | | pDR111 derivative; IPTG inducible promoter P*_hy_*___*_spank_*, *nisAT*, *spec^r^*, *amp^r^* | | This study |
| pDR111-*nisA_sfgfp_-nisT* | | pDR111 derivative; IPTG inducible promoter P*_hy_*___*_spank_*, *nisA_sfgfp_, nisT*, *spec^r^*, *amp^r^* | | This study |
| pDR111-*nisA_mKate2_-nisT* | | pDR111 derivative; IPTG inducible promoter P*_hy_*___*_spank_*, *nisA_mKate2_, nisT*, *spec^r^*, *amp^r^* | | This study |
| pDR111-*nisA-_sfgfp_nisT* | | pDR111 derivative; IPTG inducible promoter P*_hy_*___*_spank_*, *nisA, _sfgfp_nisT*, *spec^r^*, *amp^r^* | | This study |
| pDR111-*nisA-nisT_sfgfp_* | | pDR111 derivative; IPTG inducible promoter P*_hy_*___*_spank_*, *nisA, nisT_sfgfp_*, *spec^r^*, *amp^r^* | | This study |
| pDR111-*nisA-_mKate2_nisT* | | pDR111 derivative; IPTG inducible promoter P*_hy_*___*_spank_*, *nisA, _mKate2_nisT*, *spec^r^*, *amp^r^* | | This study |
| pDR111-*nisA-nisT_mKate2_* | | pDR111 derivative; IPTG inducible promoter P*_hy_*___*_spank_*, *nisA, nisT_mKate2_*, *spec^r^*, *amp^r^* | | This study |
| pDR111-*nisA_sfgfp_-_mKate2_nisT* | | pDR111 derivative; IPTG inducible promoter P*_hy_*___*_spank_*, *nisA_sfgfp_, _mKate2_nisT*, *spec^r^*, *amp^r^* | | This study |
| pDR111-*nisT_sfgfp_* | | pDR111 derivative; IPTG inducible promoter P*_hy_*___*_spank_*, *nisT_sfgfp_*, *spec^r^*, *amp^r^* | | This study |
| pDR111-*_mKate2_nisT* | | pDR111 derivative; IPTG inducible promoter P*_hy_*___*_spank_*, *_mKate2_nisT*, *spec^r^*, *amp^r^* | | This study |
| pDR111-*nisT^TMD^_sfgfp_* | | pDR111 derivative; IPTG inducible promoter P*_hy_*___*_spank_*, *nisT^TMD^_sfgfp_*, *spec^r^*, *amp^r^;* TMD, transmembrane domain | | This study |
| pDR111-*nisT^NBD^* | | pDR111 derivative; IPTG inducible promoter P*_hy_*___*_spank_*, *nisT^NBD^*, *spec^r^*, *amp^r^;* NBD, nucleotide binding domain | | This study |
| pDR111-*nisT^NBD^_sfgfp_* | | pDR111 derivative; IPTG inducible promoter P*_hy_*___*_spank_*, *nisT^NBD^_sfgfp_*, *spec^r^*, *amp^r^;* NBD, nucleotide binding domain | | This study |
| pDR111-*nisA-nisT_His_* | | pDR111 derivative; IPTG inducible promoter P*_hy_*___*_spank_*, *nisA, nisT_His_*, *spec^r^*, *amp^r^;* His, 6xHis tag | | This study |
| pDG1664-*nisB* | | pDG1664 derivative; Xylose inducible promoter P*_xylA_, nisB, ery^r^, spec^r^, amp^r^* | | This study |
| pDG1664-*nisBC* | | pDG1664 derivative; Xylose inducible promoter P*_xylA_, nisB, nisC, ery^r^, spec^r^, amp^r^* | | This study |
| pDG1664-*_sfgfp_nisB-nisC* | | pDG1664 derivative; Xylose inducible promoter P*_xylA_, _sfgfp_nisB, nisC, ery^r^, spec^r^, amp^r^* | | This study |
| pDG1664-*nisB_sfgfp_-nisC* | | pDG1664 derivative; Xylose inducible promoter P*_xylA_, nisB_sfgfp_, nisC, ery^r^, spec^r^, amp^r^* | | This study |
| pDG1664-*_mKate2_nisB-nisC* | | pDG1664 derivative; Xylose inducible promoter P*_xylA_, _mKate2_nisB, nisC, ery^r^, spec^r^, amp^r^* | | This study |
| pDG1664-*nisB_mKate2_-nisC* | | pDG1664 derivative; Xylose inducible promoter P*_xylA_, nisB_mKate2_, nisC, ery^r^, spec^r^, amp^r^* | | This study |
| pDG1664-*nisB-_sfgfp_nisC* | | pDG1664 derivative; Xylose inducible promoter P*_xylA_, nisB, _sfgfp_nisC, ery^r^, spec^r^, amp^r^* | | This study |
| pDG1664-*nisB-nisC_sfgfp_* | | pDG1664 derivative; Xylose inducible promoter P*_xylA_, nisB, nisC_sfgfp_, ery^r^, spec^r^, amp^r^* | | This study |
| pDG1664-*nisB-_mKate2_nisC* | | pDG1664 derivative; Xylose inducible promoter P*_xylA_, nisB, _mKate2_nisC, ery^r^, spec^r^, amp^r^* | | This study |
| pDG1664-*nisB-nisC_mKate2_* | | pDG1664 derivative; Xylose inducible promoter P*_xylA_, nisB, nisC_mKate2_, ery^r^, spec^r^, amp^r^* | | This study |
| pDG1664-*nisB_sfgfp_* | | pDG1664 derivative; Xylose inducible promoter P*_xylA_, nisB_sfgfp_, ery^r^, spec^r^, amp^r^* | | This study |
| pDG1664-*nisC_sfgfp_* | | pDG1664 derivative; Xylose inducible promoter P*_xylA_, nisC_sfgfp_, ery^r^, spec^r^, amp^r^* | | This study |
| pDG1664-*nisB_sfgfp_- _mKate2_nisC* | | pDG1664 derivative; Xylose inducible promoter P*_xylA_, nisB_sfgfp_, _mKate2_nisC, ery^r^, spec^r^, amp^r^* | | This study |

**References**

1. de Ruyter PG KO, de Vos WM. 1996. Controlled gene expression systems for *Lactococcus lactis* with the food-grade inducer nisin. Appl Environ Microbiol 62:3662-3667.

2. Chen JQ, Zhao LQ, Fu G, Zhou WJ, Sun YX, Zheng P, Sun JB, Zhang DW. 2016. A novel strategy for protein production using non-classical secretion pathway in *Bacillus subtilis*. Microb Cell Fact 15:69.

3. van Heel AJ, Mu DD, Montalban-Lopez M, Hendriks D, Kuipers OP. 2013. Designing and producing modified, new-to-nature peptides with antimicrobial activity by use of a combination of various lantibiotic modification enzymes. ACS Synth Biol 2:397-404.

4. Zhang XZ, Cui ZL, Hong Q, Li SP. 2005. High-level expression and secretion of methyl parathion hydrolase in *Bacillus subtilis* WB800. Appl Environ Microbiol 71:4101-4103.

5. van Tilburg AY, van Heel AJ, Stulke J, de Kok NAW, Rueff AS, Kuipers OP. 2020. Mini*Bacillus* PG10 as a convenient and effective production host for lantibiotics. ACS Synth Biol 9:1833-1842.
