# Supplementary material for: Visualization and Analysis of the Dynamic Assembly of a Heterologous Lantibiotic Biosynthesis Complex in Bacillus subtilis
Source: mBio. 2021 Jul 20;12(4):e01219-21. doi: 10.1128/mBio.01219-21 (PMC8406302; doi:10.1128/mBio.01219-21)
Supplement: TABLE S4 [file mbio.01219-21-st004.docx]

**Table S4 Oligonucleotides used in this study**

| **Primers** | **Sequence (5’--->3’)** |
| --- | --- |
| PC01 | AAATCAAAGGGGGAAATCATATGTCAAAAGGAGAAGAGCTGTTCAC |
| PC02 | CCTTCGGATCCTAGAAGCTTTTACTTATAAAGCTCATCCATGCCGTG |
| PC03 | AAATCAAAGGGGGAAATCATATGTCAGAACTTATCAAGGAAAATATGCAC |
| PC04 | CCTTCGGATCCTAGAAGCTTTTAACGGTGTCCCAATTTACTAGGC |
| PC05 | AAGCTTCTAGGATCCGAAGGCAGC |
| PC06 | ATGATTTCCCCCTTTGATTTAAGTGAACAAG |
| PC07 | AAATCAAAGGGGGAAATCATATGGATGAAGTGAAAGAATTCACATCAAAAC |
| PC08 | ATGCGGCTAGCTGTCGACTATTATTTATTCATCATTATCCTCATATTGCTCTG |
| PC09 | TAGTCGACAGCTAGCCGCATGCAAG |
| PC10 | ATGATTTCCCCCTTTGATTTAGCTTAATTGTTATCCGCTCACAATTACAC |
| PC11 | AAATCAAAGGGGGAAATCATATGAGTACAAAAGATTTTAACTTGGATTTGG |
| PC12 | CATATGTAAATCGCTCCTTTTTAGGTGTTATTTGCTTACGTGAATACTACAATGACAAG |
| PC13 | AGCAAATAACACCTAAAAAGGAGCGATTTACATATGGATGAAGTGAAAGAATTCACATC |
| PC14 | AAAGGGGGAAATCATATGATAAAAAGTTCATTTAAAGCTCAACCGTTTTTAG |
| PC15 | GGATCCTAGAAGCTTTCATTTCATGTATTCTTCCGAAACAAACAACC |
| PC16 | AAAAAGGAGCGATTTACATATGAGGATAATGATGAATAAAAAAAATATAAAAAGAAATG |
| PC17 | CCTTCGGATCCTAGAAGCTTGGTCAAAGTCATCAAACCTCTGAATTCC |
| PC18 | AAATCGCTCCTTTTTAGGTGTCATTTCATGTATTCTTCCGAAACAAACAAC |
| PC19 | GTAGCGGTGGAGGTGGCAGCATGTCAAAAGGAGAAGAGCTGTTCAC |
| PC20 | TATGTAAATCGCTCCTTTTTAGGTGTTACTTATAAAGCTCATCCATGCCGTGAG |
| PC21 | AAAAAGGAGCGATTTACATATGGATGAAGTGAAAGAATTCACATCAAAAC |
| PC22 | GCTGCCACCTCCACCGCTACCACGTCCTTCAATTTTGCTTACGTGAATACTACAATGAC |
| PC23 | GTAGCGGTGGAGGTGGCAGCATGTCAGAACTTATCAAGGAAAATATGCAC |
| PC24 | ATGTAAATCGCTCCTTTTTAGGTGTTAACGGTGTCCCAATTTACTAGGCAAATC |
| PC25 | TAAAAAGGAGCGATTTACATATGTCAAAAGGAGAAGAGCTGTTCAC |
| PC26 | CTGCCACCTCCACCGCTACCTTACTTATAAAGCTCATCCATGCCGTG |
| PC27 | GTAGCGGTGGAGGTGGCAGCATGGATGAAGTGAAAGAATTCACATCAAAAC |
| PC28 | CATATGTAAATCGCTCCTTTTTAGGTGTTATTTGCTTACGTGAATACTACAATGACAAG |
| PC29 | GGTAGCGGTGGAGGTGGCAGCATGTCAAAAGGAGAAGAGCTGTTCAC |
| PC30 | ATGCGGCTAGCTGTCGACTATTATTTACTTATAAAGCTCATCCATGCCGTG |
| PC31 | CTGCCACCTCCACCGCTACCTTCATCATTATCCTCATATTGCTCTGAATAATAAAGTTC |
| PC32 | TAAAAAGGAGCGATTTACATATGTCAGAACTTATCAAGGAAAATATGCAC |
| PC33 | GCTGCCACCTCCACCGCTACCACGGTGTCCCAATTTACTAGGCAAATC |
| PC34 | GTAGCGGTGGAGGTGGCAGCATGTCAGAACTTATCAAGGAAAATATGCAC |
| PC35 | ATGCGGCTAGCTGTCGACTATTATTTAACGGTGTCCCAATTTACTAGGCAAATC |
| PC36 | AAAGGGGGAAATCATATGGATGAAGTGAAAGAATTCACATCAAAAC |
| PC37 | ATGATTTCCCCCTTTGATTTAGCTTAATTGTTATCCGCTCACAATTACAC |
| PC38 | TAAAAAGGAGCGATTTACATATGTCAGAACTTATCAAGGAAAATATGCAC |
| PC39 | AATCGCTCCTTTTTAGGTGAGCTTAATTGTTATCCGCTCACAATTACAC |
| PC40 | GTAGCGGTGGAGGTGGCAGCATGTCAAAAGGAGAAGAGCTGTTCAC |
| PC41 | CACCTCCACCGCTACCATCTTCTATTTTTTTGTGAACTACACTTTCTCTC |
| PC42 | AAAGGGGGAAATCATATGACTGAAATATGCAATCAACATATAGGAACTG |
| PC43 | ATGATTTCCCCCTTTGATTTAGCTTAATTGTTATCCGCTCACAATTACAC |
| PC44 | CATCACCATCACCATTAAATAATAGTCGACAGCTAGCCGCATG |
| PC45 | TAATGGTGATGGTGATGATGTTCATCATTATCCTCATATTGCTCTGAATAATAAAG |
| PC46 | AAATCAAAGGGGGAAATCATATGTCAAAAGGAGAAGAGCTGTTCAC |
| PC47 | GCTGCCACCTCCACCGCTACCTTACTTATAAAGCTCATCCATGCCGTGAG |
| PC48 | GTAGCGGTGGAGGTGGCAGCATAAAAAGTTCATTTAAAGCTCAACCGTTTTTAG |
| PC49 | ATGATTTCCCCCTTTGATTTAAGTGAACAAG |
| PC50 | GTAGCGGTGGAGGTGGCAGCATGTCAAAAGGAGAAGAGCTGTTCAC |
| PC51 | TATGTAAATCGCTCCTTTTTAGGTGTTACTTATAAAGCTCATCCATGCCGTGAG |
| PC52 | AAAAAGGAGCGATTTACATATGAGGATAATGATGAATAAAAAAAATATAAAAAGAAATG |
| PC53 | GCTGCCACCTCCACCGCTACCTTTCATGTATTCTTCCGAAACAAACAACC |
| PC54 | AAATCAAAGGGGGAAATCATATGTCAGAACTTATCAAGGAAAATATGCAC |
| PC55 | CTGCCACCTCCACCGCTACCACGGTGTCCCAATTTACTAGGCAAATC |
| PC56 | GGTAGCGGTGGAGGTGGCAGCATAAAAAGTTCATTTAAAGCTCAACCGTTTTTAG |
| PC57 | GTTCTGACATATGATTTCCCCCTTTGATTTAAGTGAACAAG |
| PC58 | GTAGCGGTGGAGGTGGCAGCATGTCAGAACTTATCAAGGAAAATATGCAC |
| PC59 | CTCATATGTAAATCGCTCCTTTTTAGGTGTTAACGGTGTCCCAATTTACTAGGCAAATC |
| PC60 | AAAAAGGAGCGATTTACATATGAGGATAATGATGAATAAAAAAAATATAAAAAGAAATG |
| PC61 | GCTGCCACCTCCACCGCTACCTTTCATGTATTCTTCCGAAACAAACAACC |
| PC62 | CACCTAAAAAGGAGCGATTTACATATGTCAAAAGGAGAAGAGCTGTTCAC |
| PC63 | GCTGCCACCTCCACCGCTACCCTTATAAAGCTCATCCATGCCGTGAG |
| PC64 | TAGCGGTGGAGGTGGCAGCATGAGGATAATGATGAATAAAAAAAATATAAAAAGAAATG |
| PC65 | AAATCGCTCCTTTTTAGGTGTCATTTCATGTATTCTTCCGAAACAAACAAC |
| PC66 | GTAGCGGTGGAGGTGGCAGCATGTCAAAAGGAGAAGAGCTGTTCAC |
| PC67 | TCATCAAACCTCTGAATTCCTTACTTATAAAGCTCATCCATGCCGTG |
| PC68 | GGAATTCAGAGGTTTGATGACTTTGACC |
| PC69 | GCTGCCACCTCCACCGCTACCTTTCCTCTTCCCTCCTTTCAAAAAATCG |
| PC70 | CACCTAAAAAGGAGCGATTTACATATGTCAGAACTTATCAAGGAAAATATGCAC |
| PC71 | GCTGCCACCTCCACCGCTACCACGGTGTCCCAATTTACTAGGCAAATC |
| PC72 | GTAGCGGTGGAGGTGGCAGCATGTCAGAACTTATCAAGGAAAATATGCAC |
| PC73 | TCATCAAACCTCTGAATTCCTTAACGGTGTCCCAATTTACTAGGCAAATC |
| PC74 | AAGCTTCTAGGATCCGAAGGCAGC |
| PC75 | GGATCCTAGAAGCTTTTACTTATAAAGCTCATCCATGCCGTG |
| PC76 | AAAGGGGGAAATCATATGAGGATAATGATGAATAAAAAAAATATAAAAAGAAATG |
| PC77 | ATGATTTCCCCCTTTGATTTAAGTGAACAAG |
| PC78 | GTAGCGGTGGAGGTGGCAGCATGTCAAAAGGAGAAGAGCTGTTCAC |
| PC79 | TATGTAAATCGCTCCTTTTTAGGTGTTACTTATAAAGCTCATCCATGCCGTGAG |
| PC80 | TAAAAAGGAGCGATTTACATATGTCAGAACTTATCAAGGAAAATATGCAC |
| PC81 | GCTGCCACCTCCACCGCTACCACGTCCTTCAATTTTGCTTACGTGAATACTACAATGAC |
| PC82 | GTAGCGGTGGAGGTGGCAGCATGTCAAAAGGAGAAGAGCTGTTCAC |
| PC83 | TATGTAAATCGCTCCTTTTTAGGTGTTACTTATAAAGCTCATCCATGCCGTGAG |
| PC84 | TAAAAAGGAGCGATTTACATATGTCAGAACTTATCAAGGAAAATATGCAC |
| PC85 | GCTGCCACCTCCACCGCTACCTTTCATGTATTCTTCCGAAACAAACAACC |
